# The impact of non-pharmaceutical interventions on SARS-CoV-2 transmission across 130 countries and territories

**DOI:** 10.1186/s12916-020-01872-8

**Published:** 2021-02-05

**Authors:** Yang Liu, Christian Morgenstern, James Kelly, Rachel Lowe, CMMID COVID-19 Working Group, James Munday, C. Julian Villabona-Arenas, Hamish Gibbs, Carl A. B. Pearson, Kiesha Prem, Quentin J. Leclerc, Sophie R. Meakin, W. John Edmunds, Christopher I. Jarvis, Amy Gimma, Sebastian Funk, Matthew Quaife, Timothy W. Russell, Jon C. Emory, Sam Abbott, Joel Hellewell, Damien C. Tully, Rein M. G. J. Houben, Kathleen O’Reilly, Georgia R. Gore-Langton, Adam J. Kucharski, Megan Auzenbergs, Billy J. Quilty, Thibaut Jombart, Alicia Rosello, Oliver Brady, Katherine E. Atkins, Kevin van Zandvoort, James W. Rudge, Akira Endo, Kaja Abbas, Fiona Yueqian Sun, Simon R. Procter, Samuel Clifford, Anna M. Foss, Nicholas G. Davies, Yung-Wai Desmond Chan, Charlie Diamond, Rosanna C. Barnard, Rosalind M. Eggo, Arminder K. Deol, Emily S. Nightingale, David Simons, Katharine Sherratt, Graham Medley, Stéphane Hué, Gwenan M. Knight, Stefan Flasche, Nikos I. Bosse, Petra Klepac, Mark Jit

**Affiliations:** 1 Centre for Mathematical Modelling of Infectious Diseases, London School of Hygiene & Tropical Medicine, London, UK; 2 IPM Informed Portfolio Management, London, UK; 3 Centre on Climate Change and Planetary Health, London School of Hygiene & Tropical Medicine, London, UK

**Keywords:** Non-pharmaceutical interventions, Policy evaluation, COVID-19, SARS-CoV-2, Public health intervention, Pandemic, Quantitative, Health impact assessment, Longitudinal analysis

## Abstract

**Background:**

Non-pharmaceutical interventions (NPIs) are used to reduce transmission of SARS coronavirus 2 (SARS-CoV-2) that causes coronavirus disease 2019 (COVID-19). However, empirical evidence of the effectiveness of specific NPIs has been inconsistent. We assessed the effectiveness of NPIs around internal containment and closure, international travel restrictions, economic measures, and health system actions on SARS-CoV-2 transmission in 130 countries and territories.

**Methods:**

We used panel (longitudinal) regression to estimate the effectiveness of 13 categories of NPIs in reducing SARS-CoV-2 transmission using data from January to June 2020. First, we examined the temporal association between NPIs using hierarchical cluster analyses. We then regressed the time-varying reproduction number (*R*_*t*_) of COVID-19 against different NPIs. We examined different model specifications to account for the temporal lag between NPIs and changes in *R*_*t*_, levels of NPI intensity, time-varying changes in NPI effect, and variable selection criteria. Results were interpreted taking into account both the range of model specifications and temporal clustering of NPIs.

**Results:**

There was strong evidence for an association between two NPIs (school closure, internal movement restrictions) and reduced *R*_*t*_. Another three NPIs (workplace closure, income support, and debt/contract relief) had strong evidence of effectiveness when ignoring their level of intensity, while two NPIs (public events cancellation, restriction on gatherings) had strong evidence of their effectiveness only when evaluating their implementation at maximum capacity (e.g. restrictions on 1000+ people gathering were not effective, restrictions on < 10 people gathering were). Evidence about the effectiveness of the remaining NPIs (stay-at-home requirements, public information campaigns, public transport closure, international travel controls, testing, contact tracing) was inconsistent and inconclusive. We found temporal clustering between many of the NPIs. Effect sizes varied depending on whether or not we included data after peak NPI intensity.

**Conclusion:**

Understanding the impact that specific NPIs have had on SARS-CoV-2 transmission is complicated by temporal clustering, time-dependent variation in effects, and differences in NPI intensity. However, the effectiveness of school closure and internal movement restrictions appears robust across different model specifications, with some evidence that other NPIs may also be effective under particular conditions. This provides empirical evidence for the potential effectiveness of many, although not all, actions policy-makers are taking to respond to the COVID-19 pandemic.

**Supplementary information:**

The online version contains supplementary material available at 10.1186/s12916-020-01872-8.

## Background

Coronavirus disease 2019 (COVID-19) is an infectious disease caused by severe acute respiratory syndrome coronavirus 2 (SARS-CoV-2). The virus is easily transmissible between humans, with a basic reproduction number around 2–4 depending on the setting [1, 2]. To date, no vaccine or highly effective pharmaceutical treatment exists against COVID-19. Countries have used a range of non-pharmaceutical interventions (NPIs) such as testing suspected cases followed by isolation of confirmed cases and quarantine of their contacts, physical distancing measures such as schools and workplaces closures, income support for households affected by COVID-19 and associated interventions, and domestic and international travel restrictions [3]. These interventions aim to prevent infection introduction, contain outbreaks, and reduce peak epidemic size so that healthcare systems do not become overwhelmed. However, these interventions come at a cost. Testing and contact tracing require laboratory and public health resources to be successful at scale, government subsidies affect national budgets, while physical distancing disrupts economic activities and daily life [4]. Hence, the psychological, social, and economic cost of interventions needs to be balanced against their potential effectiveness in reducing SARS-CoV-2 spread.

Modelling studies suggest that travel restrictions [5, 6], contact tracing and quarantine [7, 8], and physical distancing [9, 10] may delay SARS-CoV-2 spread, based on assumptions about how they may change transmission between individuals in populations. However, the effectiveness of such interventions depends on factors such as societal compliance (e.g. the extent to which people reduce their daily contacts following government restrictions) that are difficult to prospectively measure. Empirical evidence about the effectiveness of specific policy interventions has been limited (see Additional file 1: Table S8 for a review) [11–37]. While several countries have seen disease incidence peak and fall [38], ascribing changes in transmission to particular interventions is difficult since countries tend to impose combinations of policy changes at different levels of stringency in close temporal sequence.

Several global databases of COVID-19-related policy interventions have been compiled [39]. Here, we used the regularly updated Oxford COVID-19 Government Response Tracker (OxCGRT) [3] and conducted panel analysis to understand the association between policy interventions and time-varying reproduction numbers (*R*_*t*_), a measure of the rate of transmission of an infectious disease in a population. We also explore whether this relationship is modulated by definitions of policy interventions, temporal lags, and population characteristics in different countries.

## Methods

### Data on NPIs and *R*_*t*_

Data on COVID-19-related NPI intensity from 1 January to 22 June 2020 was extracted on 5 July 2020 from version 5 of OxCGRT, based on the codebook version 2.2 (22 April 2020) [3]. This version contains publicly available information from 178 countries and territories on 18 NPI categories. We further divided these countries and territories into seven regions according to the World Bank classification [40]. Note that these 18 NPI categories are broad, so many specific policy interventions (e.g. facial covering mandates) are not independently coded in the database. See Additional file 1: Table S1 for further metadata.

From this database, we removed (i) “miscellaneous” policies as they contained no data at the time of our data extraction; (ii) “giving international support” and “investment in vaccines” policies as they did not on face validity have a causal pathway to influence local SARS-CoV-2 transmission within the timescale of the analysis; (iii) “fiscal measures” and “emergency investment in healthcare” policies as both the start and the duration of their effect is often unclear (e.g. the announcement of an investment may be implemented weeks later; funding that is allocated may be spent over a long time); and (iv) data after 22 June 2020 because > 10% of countries and territories have missing data after this date (see Additional file 1: Figure S1) [3]. Missing data fields on or before 22 June 2020 were imputed by (a) carrying forward or backwards the next or last non-missing observation when missingness occurred at the two tails of the time-series or (b) linearly interpolating using non-missing observations when missingness does not occur at the two tails of the time series. We divided the remaining 13 policy interventions into four policy groups roughly consistent with the original database (Table 1).

**Table 1:** Thirteen types of NPIs from OxCGRT, their general categorisations, and the coding schema used in our analysis to quantify their intensity

NPI groups	Specific NPIs	Coding schema
Internal containment and closure	School closure; workplace closure; cancellation of public events; limits on gathering sizes; closure of public transport; stay-at-home requirement; internal movement restriction	*Any effort scenario*: NPIs are binary variables, considered “present” as long as any (non-zero) effort is made. *Maximum effort scenario*: NPIs are binary variables, considered “present” only if the maximum effort is made. For example, an intervention X has levels 0–3. A record at level 2 is converted to 1 under *any effort* and 0 under *maximum effort* scenarios*.*
International travel restrictions	International movement restriction	
Economic policies	Income support; debt/contract relief for households	
Health systems policies	Public information campaign; testing policy; contact tracing	

Most NPIs in the database are measured on ordinal scales that capture intensity (e.g. 0 = no contact tracing; 1 = limited contact tracing; 2 = comprehensive contact tracing). Since the intervals between categories are not necessarily equally spaced, we converted NPI history into binary variables under two scenarios: (i) *any effort scenario*: all zero records were converted to 0, and non-zero records were converted to 1, and (ii) *maximum effort scenario*: all non-maximum records were converted to 0, and all records at maximum levels were converted to 1 (see Table 1).

Transmission of SARS-CoV-2 is routinely measured using the time-varying reproduction number (*R*_*t*_), a metric which represents the mean number of secondary cases that arise from one index case. We used the median *R*_*t*_ estimates available through EpiForecasts [https://epiforecasts.io/], a publicly available repository of *R*_*t*_ estimates for many countries. The estimation process is based on reported incidence while accounting for a range of uncertainties surrounding the incubation period, the delays between symptom onset and reporting [41]. The underlying method has been detailed in Cori et al. [42]. In short, the transmission rate of an infectious disease is approximated by the ratio between new infections at time *t* and the infectious individuals at time *t − w* where *w* is the associated time window. In EpiForecasts, a weekly time window is used. This measure is expected to fall when effective NPIs reduce the rate of SARS-CoV-2 transmission. Since the effects of some NPIs may take time to become evident, we explored a range of temporal lag effects between NPI implementation and *R*_*t*_ changes.

Between 1 January and 22 June 2020, data on NPIs and *R*_*t*_ are simultaneously available for 130 countries and territories, all of which are used in the panel analysis described below.

### Understanding the temporal patterns

The effect of an NPI on *R*_*t*_ may vary over time as a result of the evolving epidemic dynamics (e.g. decreasing number of susceptibles) or time-varying factors such as public compliance (e.g. the proportion of shoppers wearing facial coverings after government mandates). To examine this effect, we split up the time series of NPIs and *R*_*t*_ values into two parts: before and after peak NPI intensity. This was a sensitivity analysis to examine the robustness of NPIs’ effectiveness in reducing COVID-19 transmission over time.

We used OxCGRT’s stringency index (SI), a combined metric of several behaviour-related NPI measures, to determine peak NPI intensity. We then fitted a Gaussian generalised additive model (GAM) with cubic splines, using SI as the response variable and date as the sole explanatory variable for each World Bank region (i.e. the predicted regional SI is informed by all stringency index time-series within it). The peak of the predicted SI splines for each region was then examined to derive an average peak across all the regions. We then constructed two time-series: (i) the *full* time series and (ii) the *truncated* time series up to the time of peak SI.

We examined temporal clustering among different NPIs to identify potential structural confounding. If two effective NPIs are temporally clustered, one may be removed due to multicollinearity, which should not by any means be interpreted as that NPI being ineffective. Similarly, if one effective and one ineffective NPI are temporally clustered, the statistical association between the effective NPI and reductions in *R*_*t*_ may create a statistical artefact whereby the ineffective NPI may also appear to be associated with reductions in *R*_*t*_. Either way, the existence of temporal clustering could cause misinterpretation of the regression results unless it is accounted for.

To investigate the temporal clustering patterns, we conducted hierarchical cluster analysis using Ward’s method [43], which minimises within-cluster variance, under the *any effort scenario* and the *maximum effort scenario*. The inputs of the hierarchical clustering process were 13 vectors (one for each NPI under consideration), with each vector element corresponding the NPI status aligned by a unique time and location. Euclidean distance was used as the distance function between each pair of NPIs, using all available data (i.e. the full time-series for each NPI). We chose this method to compare the entire time-series of the NPIs, without having to select time-series summary metrics (e.g. the timing when an NPI was implemented) a priori. We then used multi-scale bootstrapping (*n* = 10,000) to test the statistical significance of the identified clusters, defined using approximate unbiased *p* values less than 0.05 [44]. The complete implementation of this method can be found in the GitHub repository at [https://github.com/yangclaraliu/COVID19_NPIs_vs_Rt].

### Panel analyses

We used panel (or longitudinal) regression to study the association between NPI intensity and *R*_*t*_, treating the time-series of NPI intensity and *R*_*t*_ in each country as observations of an individual in a panel. We used a linear fixed effects model:

$$ {R}_{it}={\alpha}_i+\sum \beta {X}_{it}+{u}_{it} $$

where *R*_*it*_ is the time-varying reproduction number of location *i* at time *t*, *α*_*i*_ is a location-specific intercept (assumed to remain constant over the timescale of the analysis), *βX*_*it*_ represents the 13 NPIs and their corresponding coefficients, and *u*_*it*_ is the error term. The decision to use a fixed-effects model with individual intercept (as opposed to a random-effects model) was based on the results of the Durbin-Wu-Hausman test [45]. In other words, there is insufficient evidence to support a random effect model based on global data, and the effects of each NPI on *R*_*t*_ can be characterised by fixed estimators.

We investigated the appropriate temporal lag between NPI intensity and *R*_*t*_. To do this, we calculated the deviance (natural logarithm of the sum of squared residuals divided by the number of data points) assuming errors are normally distributed for temporal lags of 1 to 21 days. Smaller deviances indicate temporal lags that provide better model fits. A temporal lag of *k* days regresses on *R*_*t*_ a particular day against NPIs implemented *k* days before (i.e. *X*_*i*(*t* − *k*)_). This analysis was carried out at both the regional and global levels. Data from North America and South Asia were excluded from region-specific temporal lag analyses due to small sample sizes.

Stepwise backwards variable selection based on Akaike and Bayesian Information Criterion (AIC or BIC) was then used to choose the most parsimonious model. Beginning with the full model (13 independent variables, one for each NPI), independent variables were removed one at a time sequentially. We also validate our results using univariable analyses and a forward variable selection algorithm.

### Statistical interpretation

For both the *any effort* and the *maximum effort scenarios*, we examined a range of model specifications including (i) different variable selection criteria: AIC and BIC, (ii) different temporal lags between the timing of NPIs and changes in *R*_*t*_ (selected based on deviance from the analysis of temporal patterns), and (iii) different time series lengths: one ending on 22 June 2020 and the other truncated to 13 April 2020, when NPI intensity peaked (on average). We then defined categories of “evidence strength” behind each association according to Table 2. For example, if an NPI has significantly negative effects on *R*_*t*_ in all but one model set-up (i.e. one of model selection criteria, temporal lags, and time-series length mentioned above), that NPI is considered to have moderate strength evidence, as long as no other NPI in the same temporal cluster has significantly positive effects on *R*_*t*_. Allocating each NPI to an evidence category was done independently by two authors (YL and MJ), with differences resolved by discussion.

**Table 2:** Expert interpretation of evidence from the statistical associations of each NPI with reductions in *R*_*t*_

Evidence strength	Effect estimates	Temporal cluster
Strong	Selected and significant with intended effect signs (i.e. negative) regardless of model specifications (i.e. variable selection criteria, temporal lags, and time-series lengths).	Not in a temporal cluster with any NPI with significantly positive effect estimates.
Moderate	Selected and significant with intended effect sign (i.e. negative) in two of three model specification dimensions (i.e. variable selection criteria, temporal lags, and time-series lengths), and non-selected or non-significant in the remaining dimension.*	
Weak	Not strong or moderate	

*For the moderate category, all the model specifications that were non-significant or non-selected were examined to see if they had a value in common across one of the three criteria, e.g. all of them had a lag of 10 days. Significance was assessed using *⍺* = 0.05

### Software

All analyses were conducted using R version 4.0.0 [46], with packages “plm” and “pvclust” [47, 48]. Code is available at https://github.com/yangclaraliu/COVID19_NPIs_vs_Rt.

## Results

### Trends in NPI intensity

**Fig. 1:**
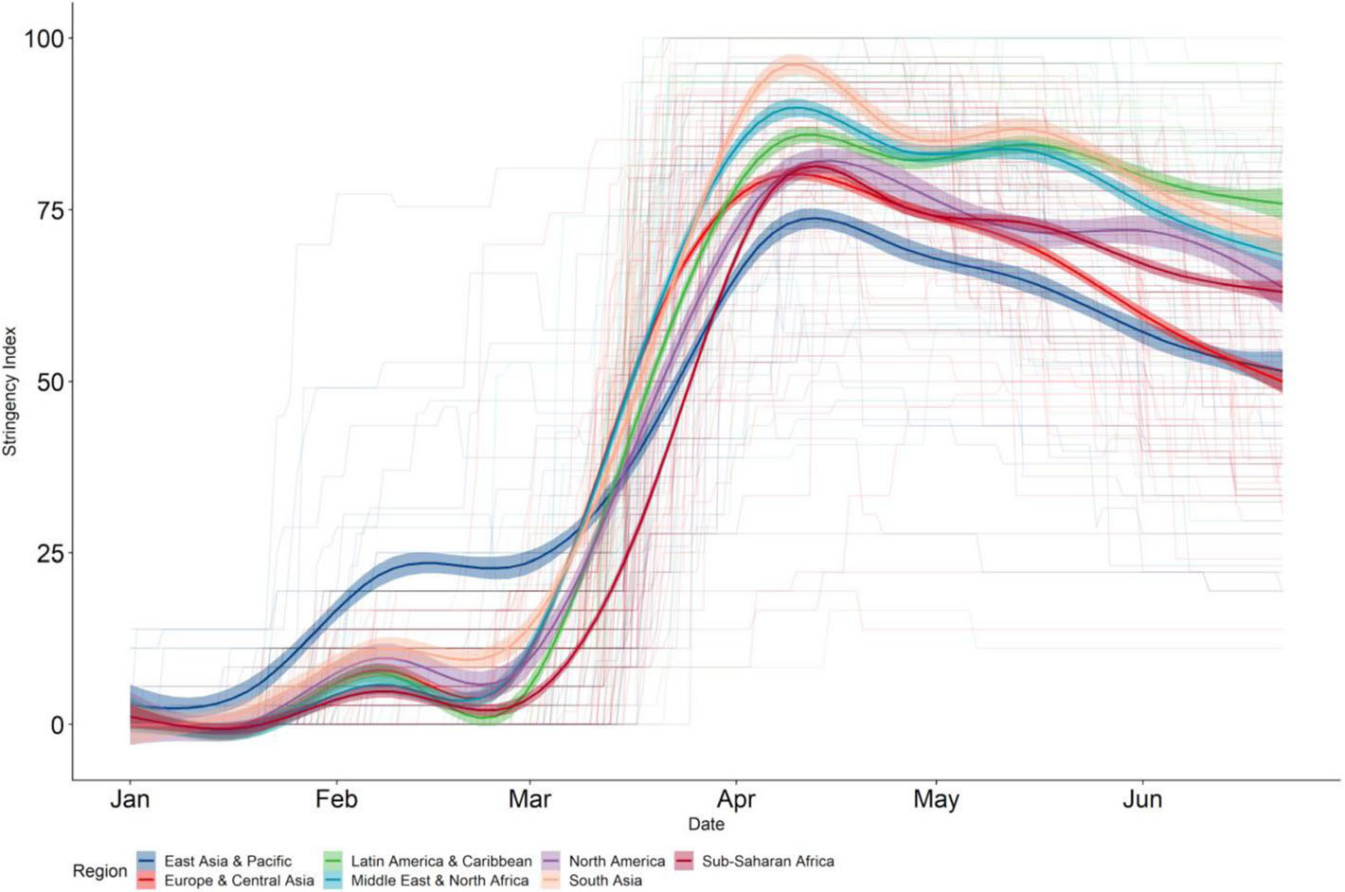
Temporal changes in NPI stringency index (range = 0-100) by region. Countries with available data are assigned corresponding geographical regions based on the World Bank classification.

Temporal trends in COVID-19-related NPI intensity measured using the OxCGRT SI are relatively consistent across regions (Fig. 1). Following the initial imposition of NPIs in China, almost all regions experienced an initial increase in policy stringency in early February 2020. The East Asia and Pacific region had the highest SI up to mid-March, but by April had the lowest SI. From March, other regions registered rapid increases in their stringency indices. The stringency index peaked in mid-April for all regions, and so 13 April 2020 was used as the time of peak NPI intensity (see Additional file 1: Table S2). All regions and nearly all countries had a higher stringency index in June compared to February.

**Fig. 2:**
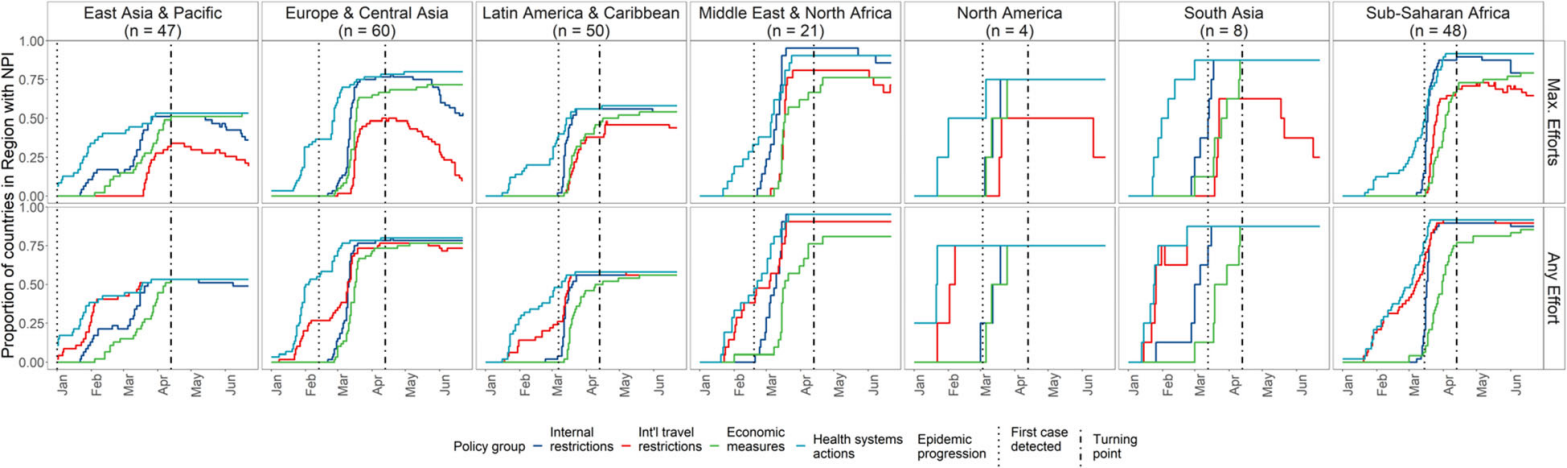
The proportions of countries implementing NPIs in each group by region. Colours indicate different NPI groups: dark blue—internal containment and closure, red—international travel restrictions, green—economic measures, and light blue—health system policies; *n* values represent the total number of countries in each World Bank region; the *y*-axis shows the proportion of countries implementing NPIs per category, e.g. one means every country in the region is implementing some measures from the NPI group. The turning point of 13 April is shown as a dotted-and-dashed vertical line.

Figure 2 shows how the intensity of specific NPI groups varies in each region relative to the time of peak intensity. Under both *any* and *maximum effort scenarios*, “Health System Policies” was the first NPI group to increase across all regions. It was also the most commonly used NPI group. This was followed by “Internal Containment and Closures” and then “Economic Policies”, although “International Travel Restrictions” sometimes came before “Internal Containment and Closures”. NPI intensity increased only as the first case was detected in each region, except for sub-Saharan Africa where many countries took action before the first detected case. While the stringency index has decreased across all regions (Fig. 1), the decreasing trends were not apparent in most NPI groups apart from International Travel Restrictions (Fig. 2).

**Fig. 3:**
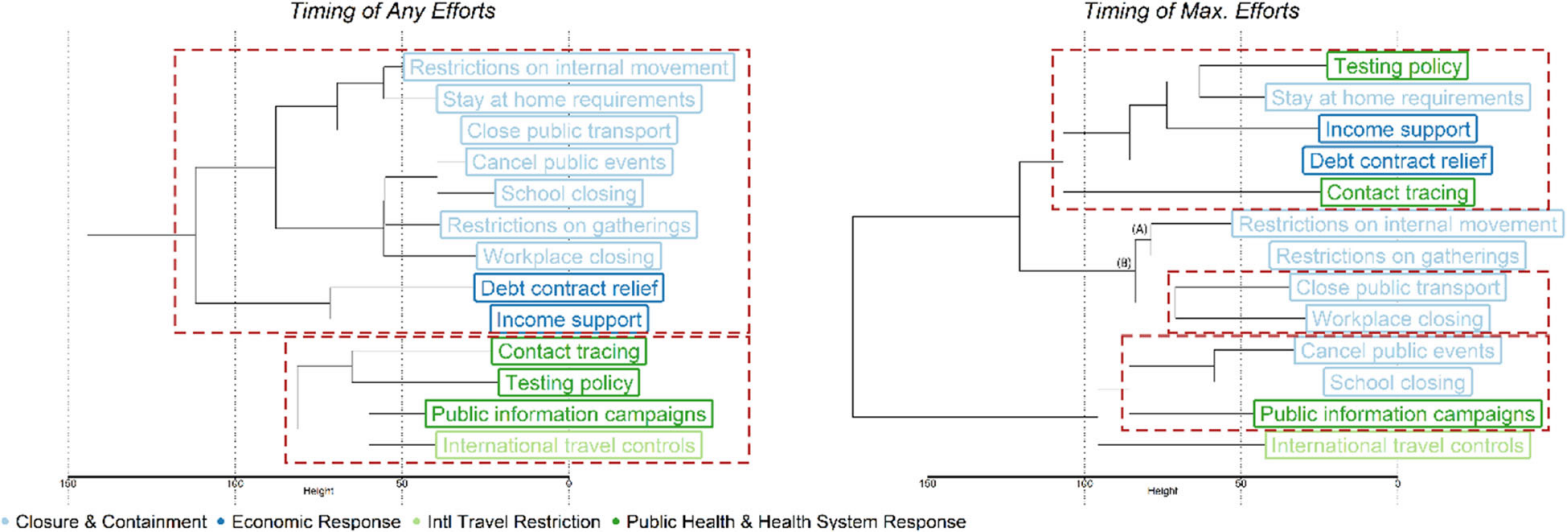
Dendrogram from the hierarchical cluster analysis of NPI time-series by scenario. The height of the node connecting two NPIs on the dendrogram represents the degree of similarity between their time-series. For example, under the *Maximum Effort Scenario*, the time-series of “restrictions on internal movement” is more similar to that of “restrictions on gatherings” (linked at point A), compared to that of “close public transport” (linked at point B). The hierarchical clustering analysis relies on Ward’s method and Euclidean distances. The colour of the text boxes corresponds to the group each NPI is in; red dashed boxes indicate statistically significantly temporal clusters identified through bootstrapping.

Hierarchical cluster analysis shows that, given the *any effort scenario*, all the NPIs are contained in two significant temporal clusters (Fig. 3). These temporal clusters align well with the broad categorisations defined in the OxCGRT, i.e. countries tend to start implementing the same categories of NPI simultaneously. However, under the *maximum effort scenario*, there are three significant temporal clusters and several NPIs are not in any cluster, i.e. countries reach their maximum level of intensity for NPIs at very different times. Specific cluster assignments are also presented in Additional file 1: Table S2–3. Direct visual representation of the association between dates when pairs of NPIs were implemented and lifted can be found in Additional file 1: Figure S4–5.

### Panel analyses

We examined the goodness-of-fit (based on deviance) of the panel regression model in all scenarios both at the regional and global level to identify the most appropriate temporal lag (see Additional file 1: Figure S4–5) [3]. For both the full and *truncated* time series (ending on 22 June and 13 April 2020, respectively), we identified temporal lags to be longest in East Asia and Pacific (between 5 and 10 days), followed by Europe and Central Asia (approximately 5 days), and the shortest in Latin America and the Caribbean (below 5 days) (see Additional file 1:Figure S4–5) [3]. The results from the Middle East and North Africa and sub-Saharan were not consistent between scenarios, and there was no clear indication of the most appropriate temporal lag when countries were all combined in a global analysis. Due to the observed heterogeneity in the temporal lags, we examined three different lag values (1, 5, and 10 days) in the regression analyses for both *full* and *truncated* time-series.

**Fig. 4:**
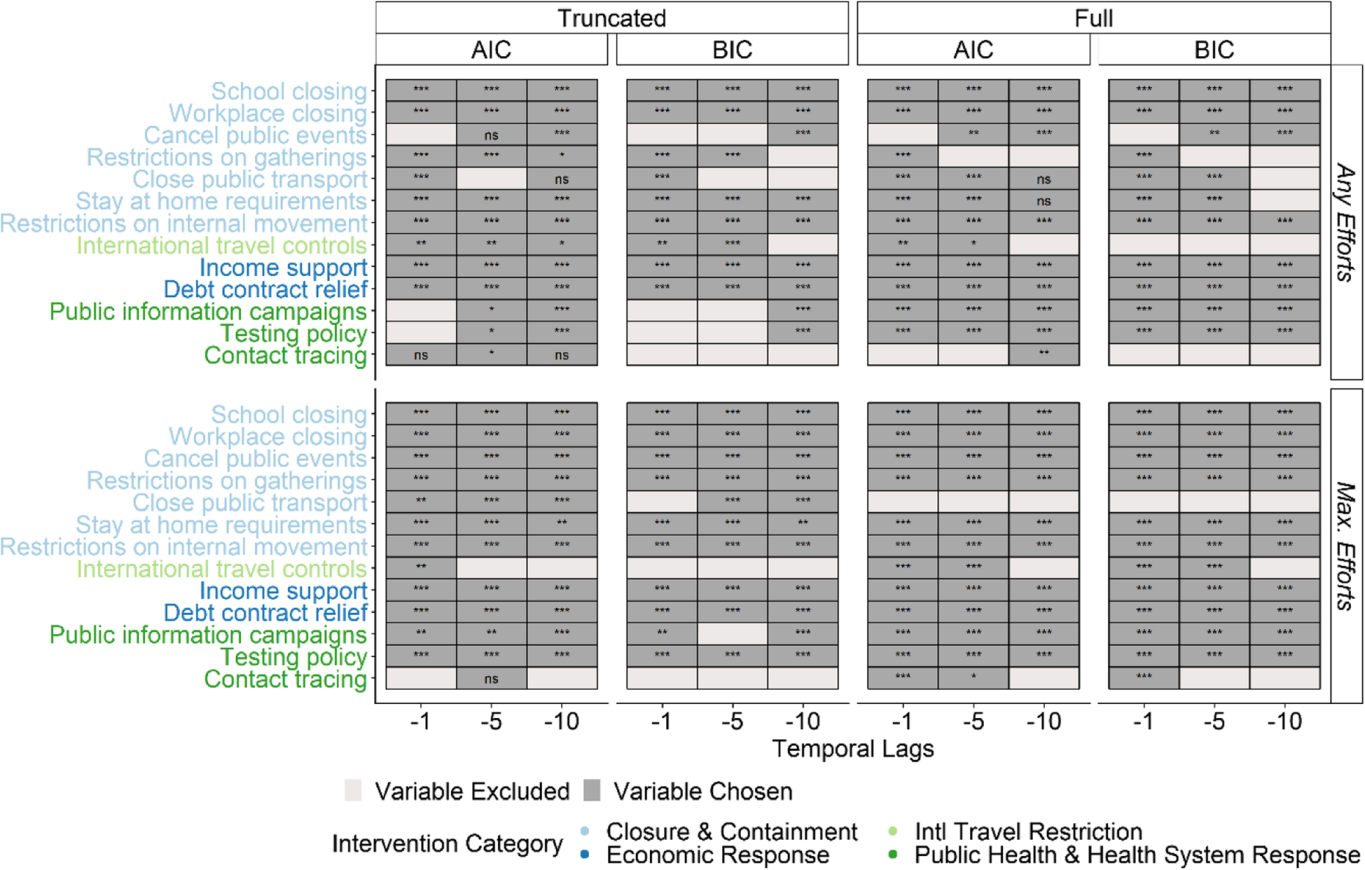
Variable selection results. Optimal models are based on a backward selection process using AIC/BIC. NPIs are colour coded based on their respective NPI categories. Cell-content represents corresponding *p* values: > 0.05—ns (i.e. non-significant); ≤ 0.05 and > 0.01—*; ≤ 0.01 and > 0.001—**; ≤ 0.001 —***.

The NPIs in the models selected based on AIC and BIC are shown in Fig. 4. Although we present the backward variable selection process in the main text under the assumption that all NPIs may explain variation in *R*_*t*_, the final models were unaltered when a forward variable selection algorithm was used. Effect validation based on univariable panel analyses can be found in Additional file 1: Figure S6–7.

Under the *any effort scenario*, the most consistently excluded NPIs were contact tracing, restrictions on gatherings, and international travel restrictions. Public information campaigns and testing policies were excluded using the *truncated* but not the *full* time-series; stay-at-home requirements were excluded using the *full* but not the *truncated* time-series. Under the *maximum effort scenario*, the most consistently excluded NPIs were contact tracing, international travel restrictions, and closure of public transportation. Public information campaigns were excluded by one model using the *truncated* time series but were always included by models using the *full* time-series. NPIs may be excluded from models either because (i) they do not affect *R*_*t*_ or (ii) their effects were fully captured by other NPIs in the same temporal clusters, and thus, they were removed by the variable selection process.

**Fig. 5:**
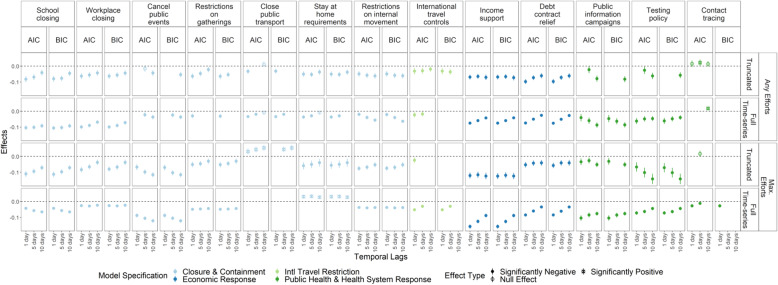
Effect sizes for each NPI from the selected models. Points and lines indicate mean and 95% confidence intervals.

Effect size estimates for the selected models in Fig. 4 are shown in Fig. 5. A few NPIs have statistically significantly positive effects: “closure of public transport”, “stay-at-home requirements”, and “contact tracing”. These results indicate that the NPIs are associated with increased *R*_*t*_. While it is not inconceivable that some NPIs may be transiently associated with increased *R*_*t*_ (e.g. increased testing efforts may be associated with increased *R*_*t*_ because they result in better detection of COVID-19 cases), variables with positive effects are likely capturing residual non-random errors for other NPIs in the same temporal cluster. Hence, they are likely biasing effect size estimates of other temporal cluster members, likely away from the null hypotheses. Hence, NPIs that are temporally related need to be interpreted within the context of the respective clusters rather than as individual measures (see also Fig. 3).

We calculated the mean absolute errors (MAE) of the final models for each country. We found that the largest residuals (i.e. worst fits) were observed in sub-Saharan Africa (highest in Zambia and Zimbabwe), East Asia and Pacific (highest in Mongolia and China), and the Middle East North Africa regions (highest in Palestine and Djibouti); the lowest residuals (i.e. best fits) were observed in sub-Saharan Africa (lowest in Namibia and Mauritania) and Latin America and the Caribbean (lowest in Colombia and El Salvador). More details can be found in Additional file 1: Table S5–6.

### Interpretation

Of the 13 NPIs in the OxCGRT, we found strong evidence for the association between two of them (school closure, internal movement restrictions) and *R*_*t*_, under both *any effort* and *maximum effort* scenarios. Another three NPIs (workplace closure, income support, and debt/contract relief) had strong evidence for an association under the *any effort* scenario only, meaning that the reductions in *R*_*t*_ were associated with the initiation of these interventions, with no evidence of greater effect as they were intensified. There was strong evidence for two other NPIs (public events cancellation, restriction on gathering) under the *maximum effort* scenario only, meaning that a reduction in *R*_*t*_ was only evident when the NPIs reached their maximum intensity.

In some cases, the sequential order in which NPIs are implemented may make it more or less likely that particular NPIs capture the effects of other NPIs or they may have interactive effects on each other (e.g. one NPI may boost or reduce the effect of a subsequent NPI). For example, the back-sampling process used in Abbott et al. [41] may attribute true *R*_*t*_ reduction to NPIs occurring later. Thus, we verify NPIs rated as being supported by strong statistical evidence by checking their sequential ordering in COVID-19 response strategies. Most of these NPIs were not implemented particularly early or late in the sequence of NPIs (Additional file 1: Figure S8–9). Complete school closure and mandatory public events cancellations are moderately left-skewed, indicating that they tend to occur first. Some (non-maximum) levels of income support and debt/contract relief are right-skewed, making it possible that their observed effects are statistical artefacts or are dependent on the imposition of earlier NPIs.

Evidence for the other NPIs was mixed. Stay-at-home requirements had moderate evidence under the *any effort* scenario, while public information campaigns had moderate evidence under the *maximum effort* scenario. Among all NPIs, some (non-maximum) levels of stay-at-home requirements tended to occur later in the overall COVID-19 response strategy. The remaining four (public transport closure, international travel controls, testing, contact tracing) had only weak evidence for an association with *R*_*t*_. Detailed interpretation of the statistics, through which these conclusions were reached, is presented in Additional file 1: Table S7. Similar methods were applied to the original raw data, without converting to *any* or *maximum effort scenarios*. However, as no statistical conclusion can be reasonably drawn, we only show the results in Additional file 1: Figure S10–11.

We observed variability in effect estimates due to differences in time-series and temporal lags used (Fig. 5). For example, the effect sizes of internal movement restrictions are smaller using the truncated time-series compared to using the full time-series. This suggests that general adherence to movement restrictions may have decreased over time. However, this variability may also be explained by the fact that full-time series include more observations. The effect sizes of public events cancellation are higher for longer temporal lags—indicating their impact on *R*_*t*_ may be delayed. These hypotheses need further validation using empirical evidence.

## Discussion

Our study used panel regression to examine the temporal association between NPIs that countries introduced in response to the COVID-19 pandemic, and its rate of transmission in populations, represented by *R*_*t*_. We explored how the association is modified according to the following model specifications: (i) level of NPI intensity (i.e. *any* vs *maximum scenarios*), (ii) model selection criteria (i.e. AIC vs BIC), (iii) varying lag effects, and (iv) different time-series lengths (i.e. truncated vs. full time-series).

We found the strength of evidence behind an association between NPIs and *R*_*t*_ depended on these model specifications. Only two NPIs (school closure, internal movement restrictions) showed unequivocal evidence of being associated with a decrease in *R*_*t*_ regardless of the assumptions made. Whether schools should stay closed has attracted debate. Keeping schools closed could potentially hurt children’s educational development and general wellbeing. Resuming schools, on the other hand, may increase COVID-19 transmission risks for both students and teachers. Our findings are consistent with much existing literature—although school closures cannot single-handedly suppress an outbreak, they are generally effective in terms of reducing transmission [49, 50].

We found evidence that internal movement restrictions reduced *R*_*t*_, but no evidence of a similar effect for international travel restrictions. The latter is consistent with Russell et al., which shows international movement restrictions have a limited impact on the epidemic dynamics of COVID-19 for most countries [51]. This difference may be explained by the types of movement interrupted—internal movement restrictions interrupt trips of all lengths whereas international movement restrictions only disrupt longer trips, which are much less common. Additionally, internal and international movement restrictions were likely used in different epidemic contexts—internal movement restrictions tend to be used more often to prevent outbreaks from escalating whereas international travel restrictions make more sense in preventing infection introduction [52]. The latter effect is not well represented in our data since *R*_*t*_ can only be estimated in settings with existing COVID-19 outbreaks (i.e. after introduction).

There are differences in the strength and direction of the effects of some NPIs (such as public transport closure and stay-at-home requirements) depending on whether the whole time series of data was used, or only data up to the date of peak NPI stringency (13 April 2020). This may indicate that these NPIs might have different effects at the start of the pandemic compared to later on, so when the NPIs were removed (likely after the peak), *R*_*t*_ did not return to its original level before the introduction of the NPIs.

The best-fitting models also support a considerable delay between NPIs and their effect on transmission. This delay is about a week on average but differs widely between regions. It could reflect delays between policies being put in place and actual behaviour change. It could also reflect delays in reporting, although these are explicitly accounted for in the *R*_*t*_ estimation in EpiForecasts—the same onset-to-delay distribution is applied in all countries [41] and hence may not reflect differences between settings. Delays of up to 3 weeks between policy changes and changes in reported cases have been documented [53].

We were not able to find evidence that supports the effectiveness of contact tracing and testing policies. This may be because both contact tracing and testing policies could lead to more cases being reported, as well as interrupting onward transmission, so the overall effect is the combination of these two opposing effects. While calculating the *R*_*t*_, EpiForecasts does not explicitly account for changes in reporting rate [41]. Another potential explanation is the way NPIs are reported in the OxCGRT, which largely relies on publicly available data sources, such as news articles. Contact tracing and test policies are both well-known public health intervention tools and have minimal impacts on the lives of those who are not potentially infected. Thus, they may be less likely to receive media coverage, compared to more disruptive NPIs such as workplace closures.

We focused our discussion on the direction and relative magnitude of the estimated effect of different NPIs, within the context of their temporal clusters during the on-going COVID-19 pandemic. The actual values of NPI-specific effect sizes, which were found to be greater for “School Closures” and “Workplace Closures” under the *any effort scenario* and for “Cancellation of Public Events” and “Income Support” under the *maximum effort scenario*, should be interpreted with caution. Given the statistical approach and the ecological design of the study, these numerical values are difficult to interpret due to structural confounding. For example, when a temporal cluster was effective in reducing *R*_*t*_, we were not able to confidently attribute the effects to particular NPIs within the cluster. As the pandemic progresses, data on more diverse NPI implementation profiles and outcomes may become available, enabling more precise determination of effect sizes.

Many other papers have explored the impact of physical distancing measures on SARS-CoV-2 transmission. Prospective mechanistic transmission models have explicitly modelled contacts relevant to viral transmission between individuals in different subgroups (e.g. ages), as well as the impact that NPIs may have on these contacts. Such studies mainly use data from a single location only such as Wuhan [9], Hong Kong [54], the USA [55], and the UK [50]. They suggest that physical distancing interventions can have a large impact on transmission. While the impact of income-related interventions has been less well studied, country reports suggest that they often play an important role in ensuring adherence to distancing measures [56].

Another group of studies have used empirical data to retrospectively examine whether NPIs have been effective in reducing transmission, using either statistical methods or mechanistic epidemiological models. Many such studies look at single interventions such as travel restrictions [25] or “lockdowns” [22, 27]. Therefore, they are less useful to policy-makers wanting to establish which of a basket of NPIs are most effective.

Only a small number have looked at multiple interventions across multiple countries (see Additional File 1: Table S8 for a review). These relate NPIs from databases to proxies of transmission such as *R*_*t*_ estimated from cases and/or deaths [18, 21, 37], or the rate of change in cases directly [13, 16, 57]. Our work demonstrates the major challenges that all such studies (including ours) face—NPI introductions are highly correlated in time, so it is difficult to independently identify the effect of each NPI due to structural confounding. A few studies partially account for this using techniques such as examining whether the number of NPIs that had already been implemented affects the impact of subsequent NPIs [11] or excluding statistically non-significant variables after all NPIs are included initially [12].

Our study extends previous work to address this problem in several ways. Firstly, we use data across a larger number of countries and territories and longer time series (January–June 2020), enhancing the power to detect independent effects even when there is partial collinearity. Second, instead of assuming that all NPIs tested have an effect like previous work, we conduct variable selection to identify only those NPIs that are retained in parsimonious models. Third, we conduct cluster analysis to explicitly identify temporal correlations, and use this in our interpretation of the strength of evidence behind each intervention. Fourth, we have conducted sensitivity analyses across a range of model specifications around the variable selection criteria, temporal lag between NPIs and change in transmission, temporal truncation, and the way NPI intensity is coded.

Nonetheless, our study also has several limitations. First, besides the information bias in the NPIs database discussed above, the coding scheme may also introduce potential bias. For example, NPIs coded as “comprehensive contact tracing for all identified cases” may have different implications in different countries. Effectiveness of contact tracing in places like Singapore [58] may be masked by seemingly similar but realistically non-comparable contact tracing programmes elsewhere. Second, compared to daily incidence, *R*_*t*_ estimates are much more suitable for cross-country comparisons and thus are used as the metric for COVID-19 transmission in this study. However, these estimates are based on a series of assumptions that may not always be appropriate. For example, the underlying methods assume constant case ascertainment rates over the 12-week time window (March–June 2020) over which our analysis takes place. Consequently, declines in *R*_*t*_ over time may have been obscured by improvements in case ascertainment, leading to some effective NPIs appearing ineffective in our analyses. We have partially adjusted for this by giving weight in our interpretation only to NPIs whose effect direction is robust to changes in the time-series length. Another limitation is that our model also does not propagate uncertainty around *R*_*t*_ estimates. Third, although we examined a wide range of NPIs, we did not include any potential interactions in the current model. Such interaction is a possibility, e.g. more people may comply with workplace closures when receiving income support. Future research should look into these relationships. Last but not the least, although OxCGRT is one of the most comprehensive databases of COVID-19-related NPIs to our knowledge, it does not capture individual behaviour such as face-covering use in public spaces. Thus, we were not able to assess the effectiveness of such measures in reducing COVID-19. Such behavioural measures may prove crucial to controlling COVID-19 epidemics, so analyses of datasets that capture adherence to these measures (e.g. survey of public behaviours [59]) may yield important insights in the future.

## Conclusions

In conclusion, evidence from a panel of 130 countries and territories provides evidence about the effectiveness of school closure and internal movement restrictions in reducing SARS-CoV-2 transmission. Despite the inherent limitations of observational and ecological data, our study provides the broadest empirical evaluation on the relative effectiveness of NPI in reducing COVID-19 transmission, while addressing the issue of structural confounding due to temporal clustering.

## Supplementary Information

**Additional file 1.** **Table S1:** Metadata on Policy Code in the Oxford COVID-19 Government Response Tracker. **Table S2:** Peak timing of stringency indices by region. **Table S3:** Results of hierarchical clustering of time-series using the any effort scenario. **Table S4:** Results of hierarchical clustering of time-series using the maximum effort scenario. **Table S5:** Lowest performance model fit by country. **Table S6:** Highest performance model fit by country. **Table S7:** Statistical interpretation worksheets. **Table S8:** Review of existing literature. **Figure S1:** The number of countries and regions with available data in the Oxford COVID-19 Government Response Tracker. **Figure S2:** The pair-wise scatter plot of NPI timing under the Any Effort Scenario. **Figure S3:** The pair-wise scatter plot of NPI timing under the Maximum Effort Scenario. **Figure S4:** Deviance from panel analyses using different temporal lags between effective reproduction number and policy interventions based on full time-series. **Figure S5:** Deviance from panel analyses using different temporal lags between effective reproduction number and policy interventions based on the truncated time-series. **Figure S6:** Univariable panel analyses – effect sizes. **Figure S7:** Univariable panel analyses – p-values. **Figure S8:** The sequential order of different NPIs under any effort scenario. **Figure S9:** The sequential order of different NPIs under maximum effort scenario. **Figure S10:** Hierarchical cluster analysis of NPIs time-series using the multilevel scenario. **Figure S11:** Effect sizes for each NPI from the selected models based on multilevel scenario.

## Data Availability

This study relies entirely on data that are either publically available or from published literature. Code used can be found at [https://github.com/yangclaraliu/COVID19_NPIs_vs_Rt].
