# Supplementary material for: The impact of non-pharmaceutical interventions on SARS-CoV-2 transmission across 130 countries and territories
Source: BMC Med. 2021 Feb 5;19:40. doi: 10.1186/s12916-020-01872-8 (PMC7861967; doi:10.1186/s12916-020-01872-8)
Supplement: Supplementary file 1 — Additional file 1. Table S1: Metadata on Policy Code in the Oxford COVID-19 Government Response Tracker. Table S2: Peak timing of stringency indices by region. Table S3: Results of hierarchical clustering of time-series using the any effort scenario. Table S4: Results of hierarchical clustering of time-series using the maximum effort scenario. Table S5: Lowest performance model fit by country. Table S6: Highest performance model fit by country. Table S7: Statistical interpretation worksheets. Table S8: Review of existing literature. Figure S1: The number of countries and regions with available data in the Oxford COVID-19 Government Response Tracker. Figure S2: The pair-wise scatter plot of NPI timing under the Any Effort Scenario. Figure S3: The pair-wise scatter plot of NPI timing under the Maximum Effort Scenario. Figure S4: Deviance from panel analyses using different temporal lags between effective reproduction number and policy interventions based on full time-series. Figure S5: Deviance from panel analyses using different temporal lags between effective reproduction number and policy interventions based on the truncated time-series. Figure S6: Univariable panel analyses – effect sizes. Figure S7: Univariable panel analyses – p-values. Figure S8: The sequential order of different NPIs under any effort scenario. Figure S9: The sequential order of different NPIs under maximum effort scenario. Figure S10: Hierarchical cluster analysis of NPIs time-series using the multilevel scenario. Figure S11: Effect sizes for each NPI from the selected models based on multilevel scenario. [file 12916_2020_1872_MOESM1_ESM.docx]

[Additional file 1] The impact of non-pharmaceutical interventions on SARS-CoV-2 transmission across 130 countries and territories

Yang Liu^1, ^^, Christian Morgenstern^2, ^^, James Kelly^2^, Rachel Lowe^1, 3^, CMMID COVID-19 Working Group, Mark Jit^1, *^

^1^Centre for Mathematical Modelling of Infectious Disease, London School of Hygiene & Tropical Medicine, London, United Kingdom

^2^ IPM Informed Portfolio Management, United Kingdom

^3^ Centre on Climate Change and Planetary Health, London School of Hygiene & Tropical Medicine, Lonodn, United Kingdom

^^^ These authors contributed equally

* Corresponding author: [mark.jit@lshtm.ac.uk](mailto:mark.jit@lshtm.ac.uk)

The following authors were part of the Centre for Mathematical Modelling of Infectious Disease COVID-19 Working Group, who contributed to providing valuable comments/ feedback to this work: James Munday; C Julian Villabona-Arenas; Hamish Gibbs; Carl A B Pearson; Kiesha Prem; Quentin J Leclerc; Sophie R Meakin; W John Edmunds; Christopher I Jarvis; Amy Gimma; Sebastian Funk; Matthew Quaife; Timothy W Russell; Jon C Emery; Sam Abbott; Joel Hellewell; Damien C Tully; Rein M G J Houben; Kathleen O’Reilly; Georgia R Gore-Langton; Adam J Kucharski; Megan Auzenbergs; Billy J Quilty; Thibaut Jombart; Alicia Rosello; Oliver Brady; Katherine E. Atkins; Kevin van Zandvoort; James W Rudge; Akira Endo; Kaja Abbas; Fiona Yueqian Sun; Simon R Procter; Samuel Clifford; Anna M Foss; Nicholas G. Davies; Yung-Wai Desmond Chan; Charlie Diamond; Rosanna C Barnard; Rosalind M Eggo; Arminder K Deol; Emily S Nightingale; David Simons; Katharine Sherratt; Graham Medley; Stéphane Hué; Gwenan M Knight; Stefan Flasche; Nikos I Bosse; Petra Klepac

# **A1. Metadata on the OxCGRT**

For visualisation, in the technical appendix, NPI related metrics are shown by their NPI code – not their full name. More detailed categorisations in each NPI group can be found at [https://github.com/OxCGRT/covid-policy-tracker/blob/master/documentation/codebook.md].

**Table S1.** Metadata on Policy Code in the Oxford COVID-19 Government Response Tracker

| NPI Group | NPI Code | Specific NPIs |
| --- | --- | --- |
| Internal containment and closure | C1 | School Closures |
|  | C2 | Workplace Closure |
|  | C3 | Cancellation of Public Events |
|  | C4 | Limits on Gathering Sizes |
|  | C5 | Closure of Public Transport |
|  | C6 | Stay-at-home Requirement |
|  | C7 | Internal Movement Requirement |
| International Travel Restrictions | C8 | International Movement Restrictions |
| Economic Policies | E1 | Income Support |
|  | E2 | Debt/ Contract Relief for Households |
| Health System Policies | H1 | Public Information Campaign |
|  | H2 | Testing Policy |
|  | H3 | Contact Tracing |

# **A2. Missing-ness in data**

The figure below shows the number of countries and regions each day between 1 January - 5 July 2020 with at least one non-missing entry in Oxford COVID-19 Government Response Tracker [[3]](https://www.zotero.org/google-docs/?UXmBt0). The vertical solid line indicates the cut-off threshold of 22 June 2020 used in this study.


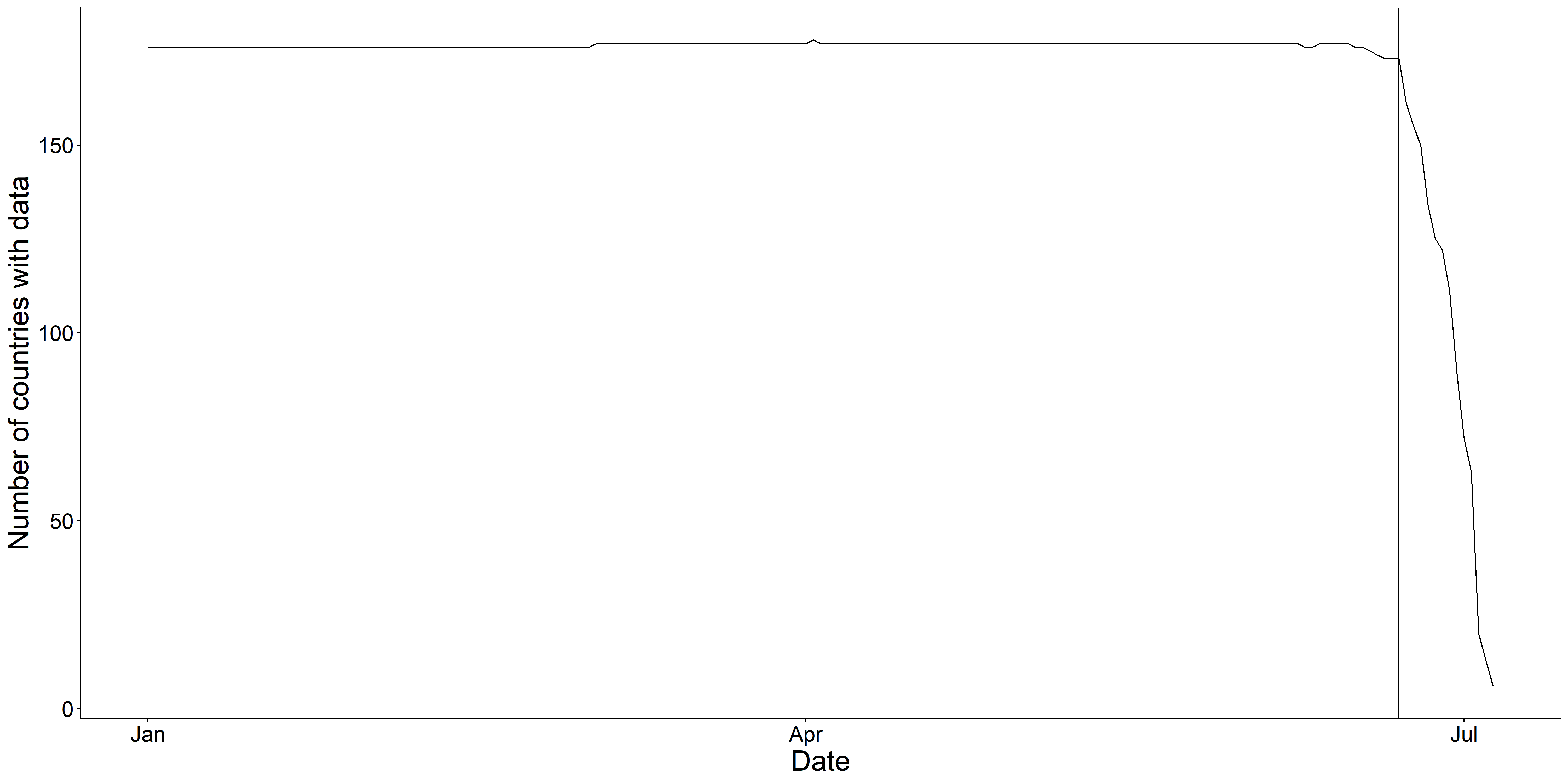


**Figure S1.** The number of countries and regions with available data in the Oxford COVID-19 Government Response Tracker.

# **A3. Peak timing of stringency indices by region**

| **Region** | **Peak Date** |
| --- | --- |
| East Asia & Pacific | 2020-04-13 |
| Europe & Central Asia | 2020-04-11 |
| Latin America & Caribbean | 2020-04-13 |
| The Middle East & North Africa | 2020-04-11 |
| North America | 2020-04-15 |
| South Asia | 2020-04-11 |
| Sub-Saharan Africa | 2020-04-13 |

**Table S2.** Peak timing of stringency indices by region.

* Peak date is defined as the date corresponding to the highest predicted stringency index on the regional level based on the general additive models.

**A4. Temporal Clusters Identified**

***Any Effort* *Scenario***

| NPI Group | NPI Code | Specific NPIs | Cluster # |
| --- | --- | --- | --- |
| Internal containment and closure | C1 | School Closures | 2 |
|  | C2 | Workplace Closure | 2 |
|  | C3 | Cancellation of Public Events | 2 |
|  | C4 | Limits on Gathering Sizes | 2 |
|  | C5 | Closure of Public Transport | 2 |
|  | C6 | Stay-at-home Requirement | 2 |
|  | C7 | Internal Movement Requirement | 2 |
| International Travel Restrictions | C8 | International Movement Restrictions | 1 |
| Economic Policies | E1 | Income Support | 2 |
|  | E2 | Debt/ Contract Relief for Households | 2 |
| Health System Policies | H1 | Public Information Campaign | 1 |
|  | H2 | Testing Policy | 1 |
|  | H3 | Contact Tracing | 1 |

**Table S3.** Results of hierarchical clustering of time-series using the *any effort scenario*.

***Maximum Effort* *Scenario***

| NPI Group | NPI Code | Specific NPIs | Cluster # |
| --- | --- | --- | --- |
| Internal containment and closure | C1 | School Closures | 2 |
|  | C2 | Workplace Closure | 1 |
|  | C3 | Cancellation of Public Events | 2 |
|  | C4 | Limits on Gathering Sizes | NA |
|  | C5 | Closure of Public Transport | 1 |
|  | C6 | Stay-at-home Requirement | 3 |
|  | C7 | Internal Movement Requirement | NA |
| International Travel Restrictions | C8 | International Movement Restrictions | NA |
| Economic Policies | E1 | Income Support | 3 |
|  | E2 | Debt/ Contract Relief for Households | 3 |
| Health System Policies | H1 | Public Information Campaign | 2 |
|  | H2 | Testing Policy | 3 |
|  | H3 | Contact Tracing | 3 |

**Table S4.** Results of hierarchical clustering of time-series using the *maximum effort scenario*.

# **A5. Pair-wise Scatter Plots Comparing the Timing of Policy Change**

***Any Effort Scenario***


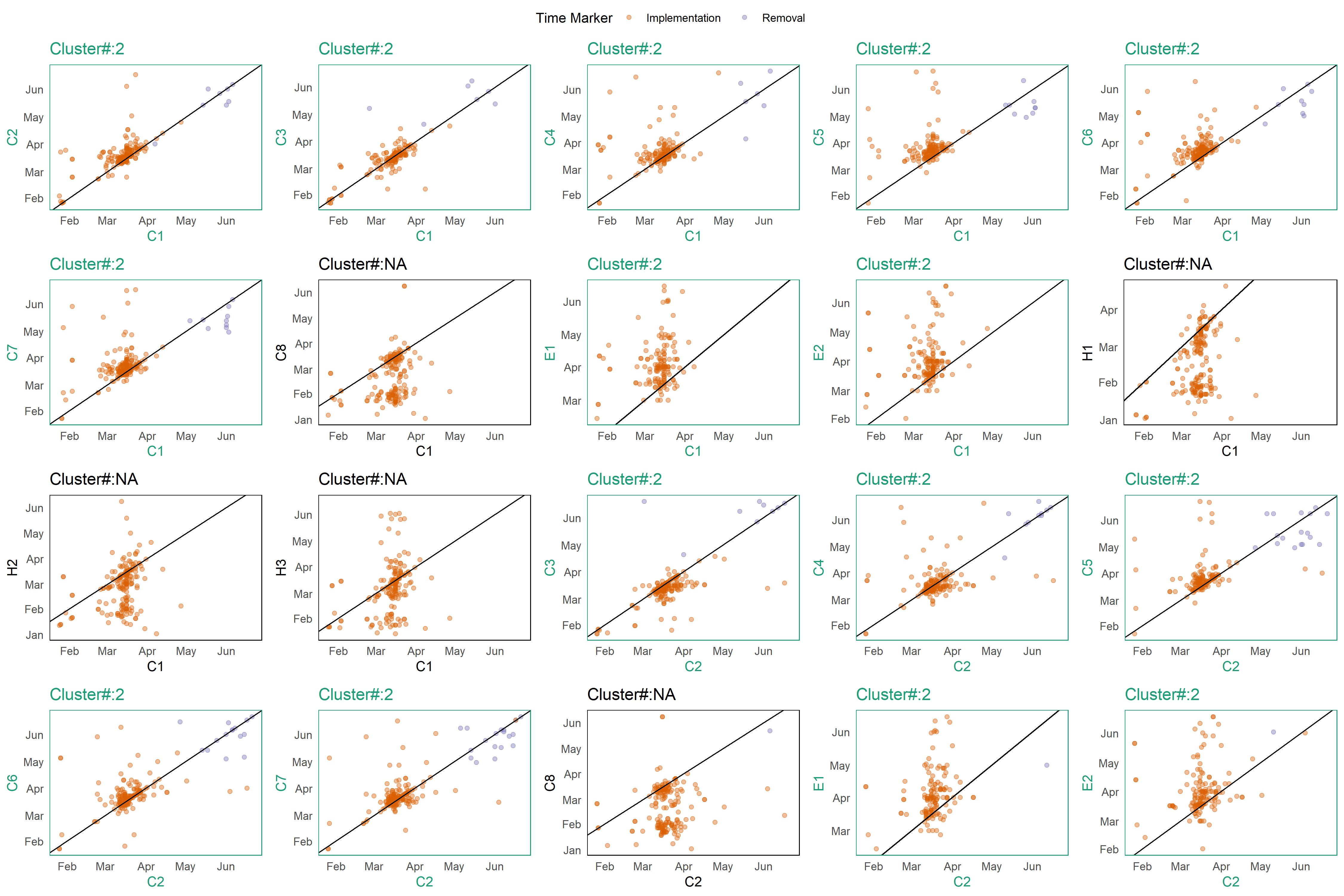


Figure S2. The pair-wise scatter plot of NPI timing under the *Any Effort* Scenario. Orange represents the timing of NPI implementation (when the corresponding variable changed from 0 to 1); purple represents the timing of lifting NPI (when the corresponding variable changed from 1 to 0). Green labels indicate pairs of NPIs identified by the hierarchical clustering analysis and the subsequent bootstrapping procedure. These specific clusters are also presented in the corresponding section on *Any Effort* Scenario in A4.

***Any Effort Scenario***


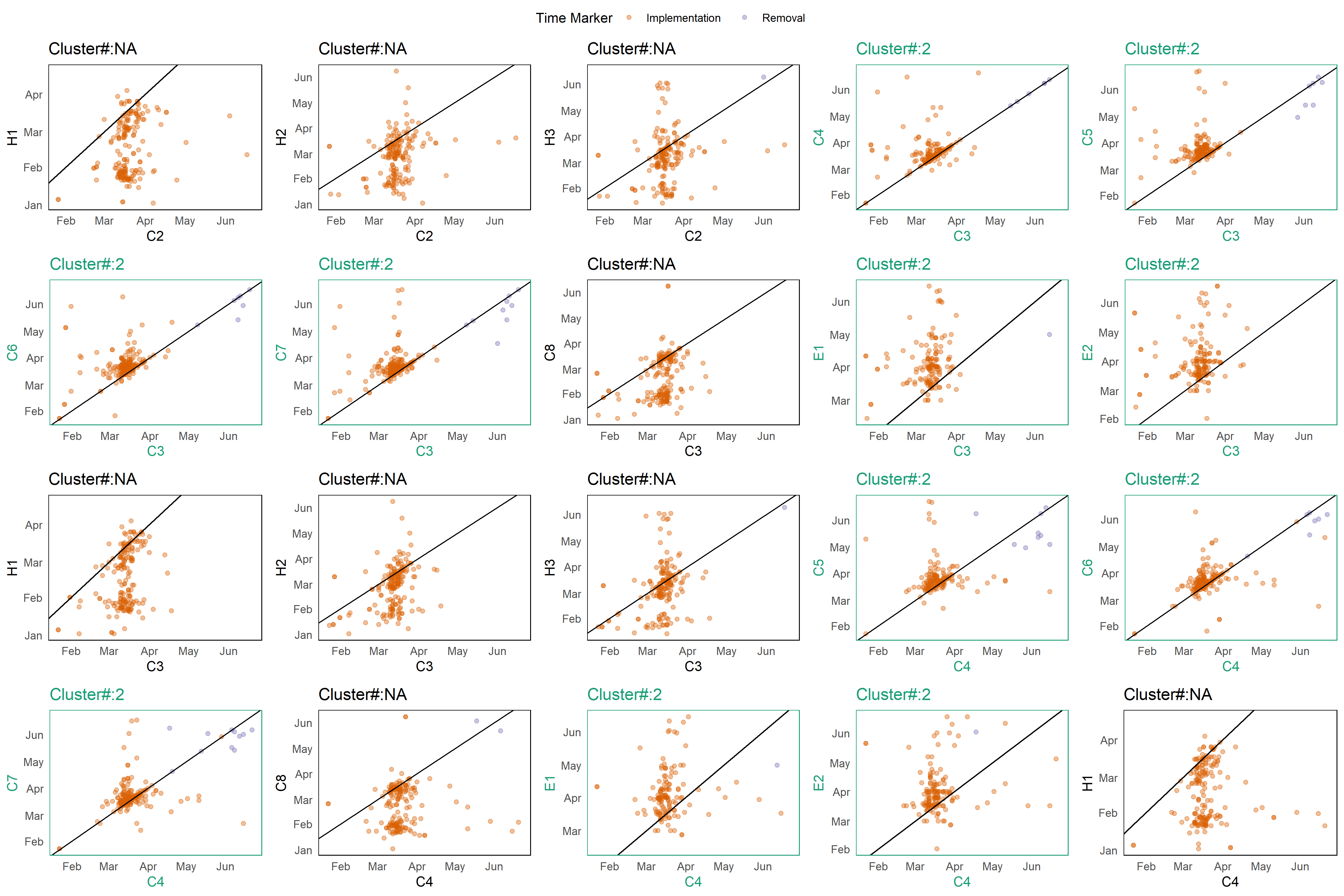


Figure S2 (continued).

***Any Effort Scenario***


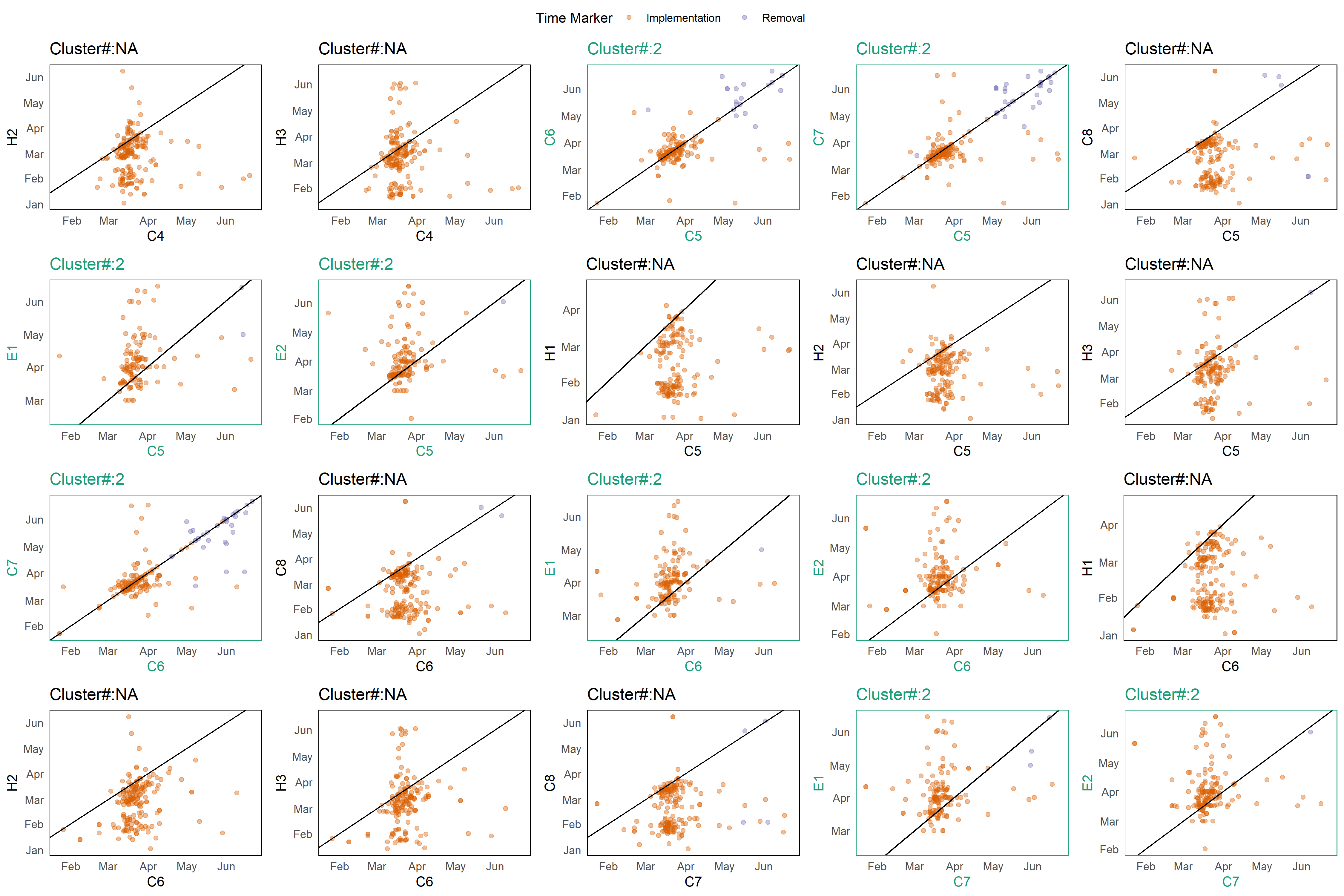


Figure S2 (continued).

***Any Effort Scenario***


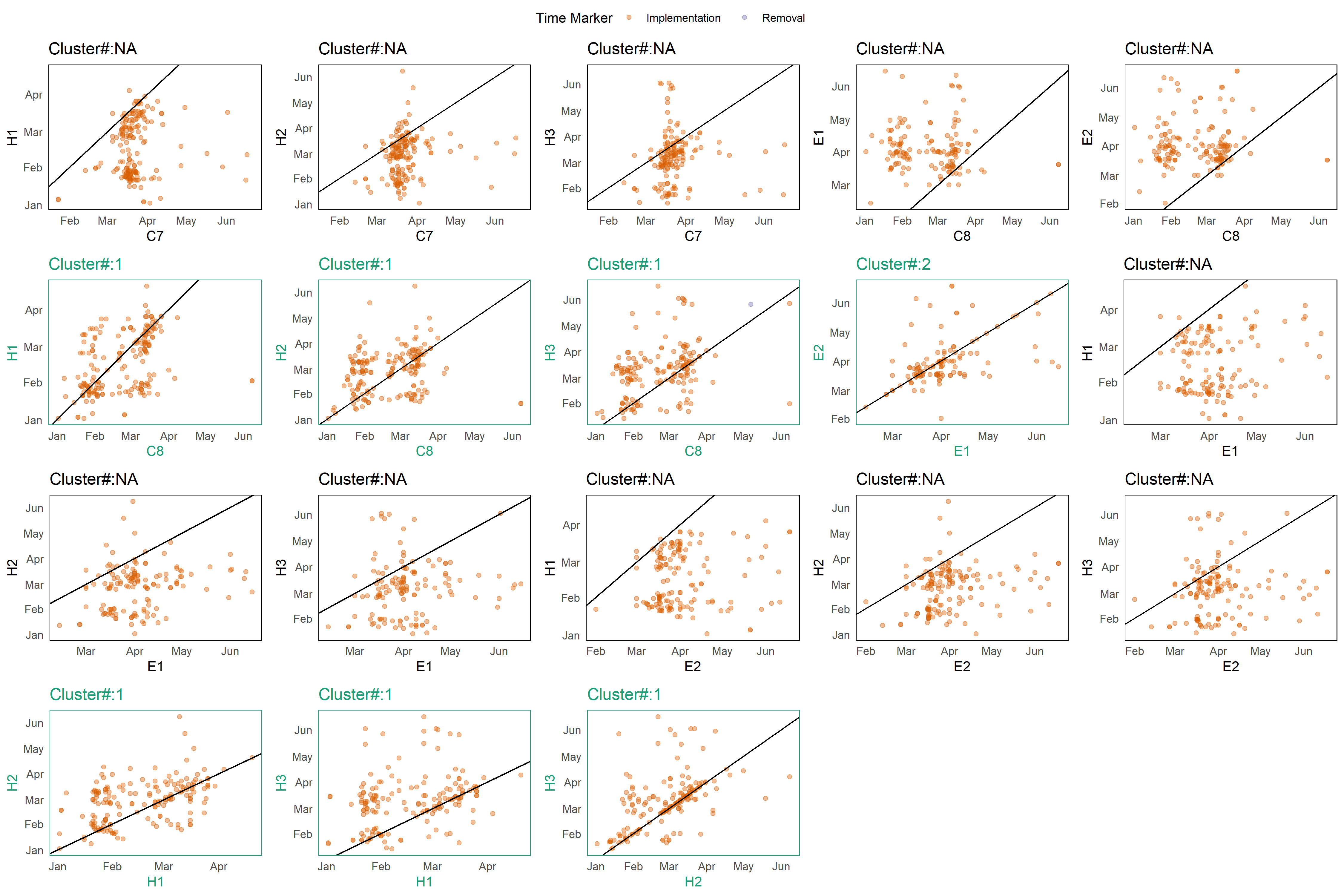


Figure S2 (continued).

***Maximum Effort Scenario***


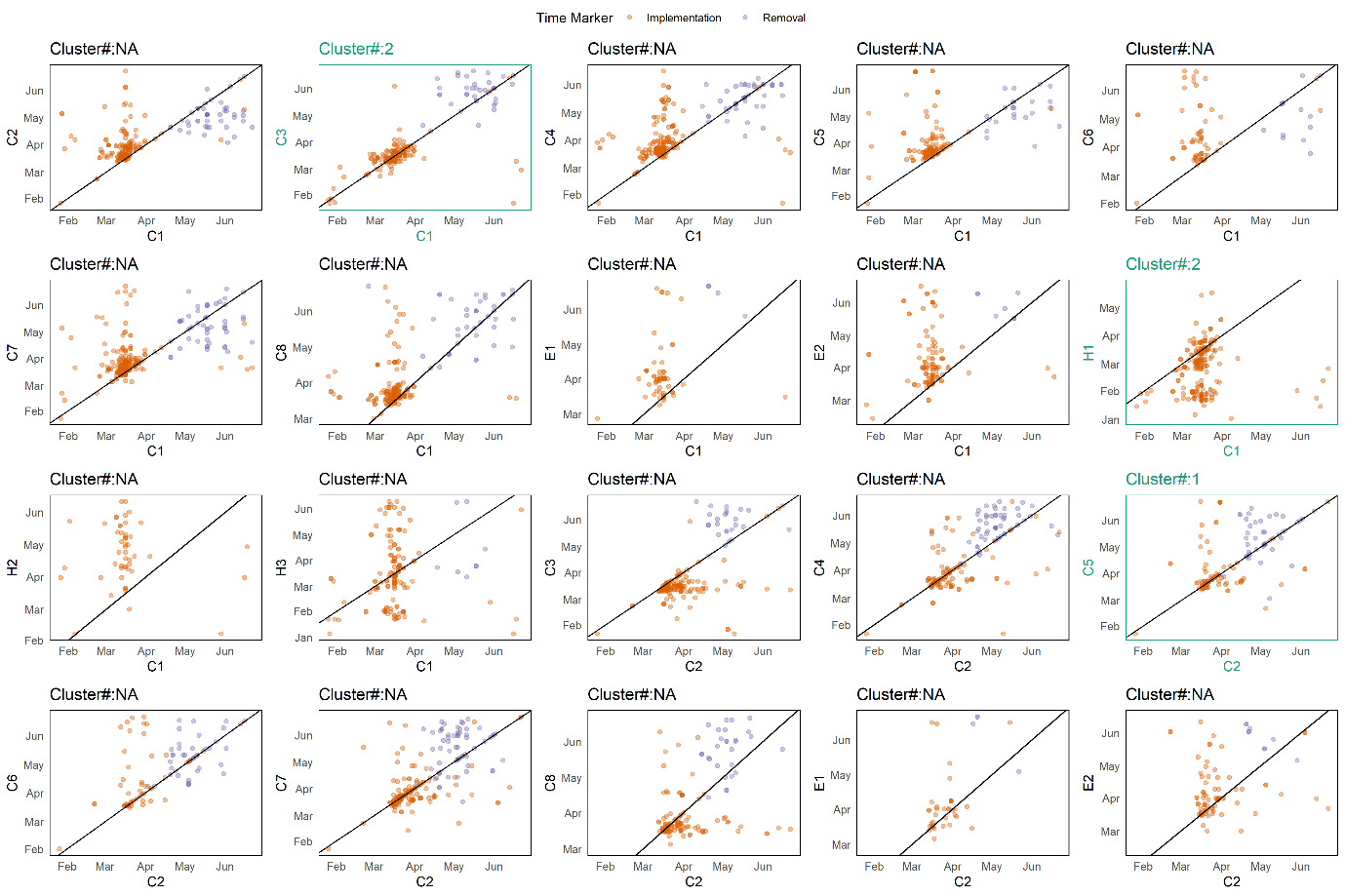


Figure S3. The pair-wise scatter plot of NPI timing under the *Maximum Effort* Scenario. Orange represents the timing of NPI implementation (when the corresponding variable changed from 0 to 1); purple represents the timing of lifting NPI (when the corresponding variable changed from 1 to 0). Green labels indicate pairs of NPIs identified by the hierarchical clustering analysis and the subsequent bootstrapping procedure. These specific clusters are also presented in the corresponding section on *Maximum Effort* Scenario in A4.

***Maximum Effort Scenario***

**
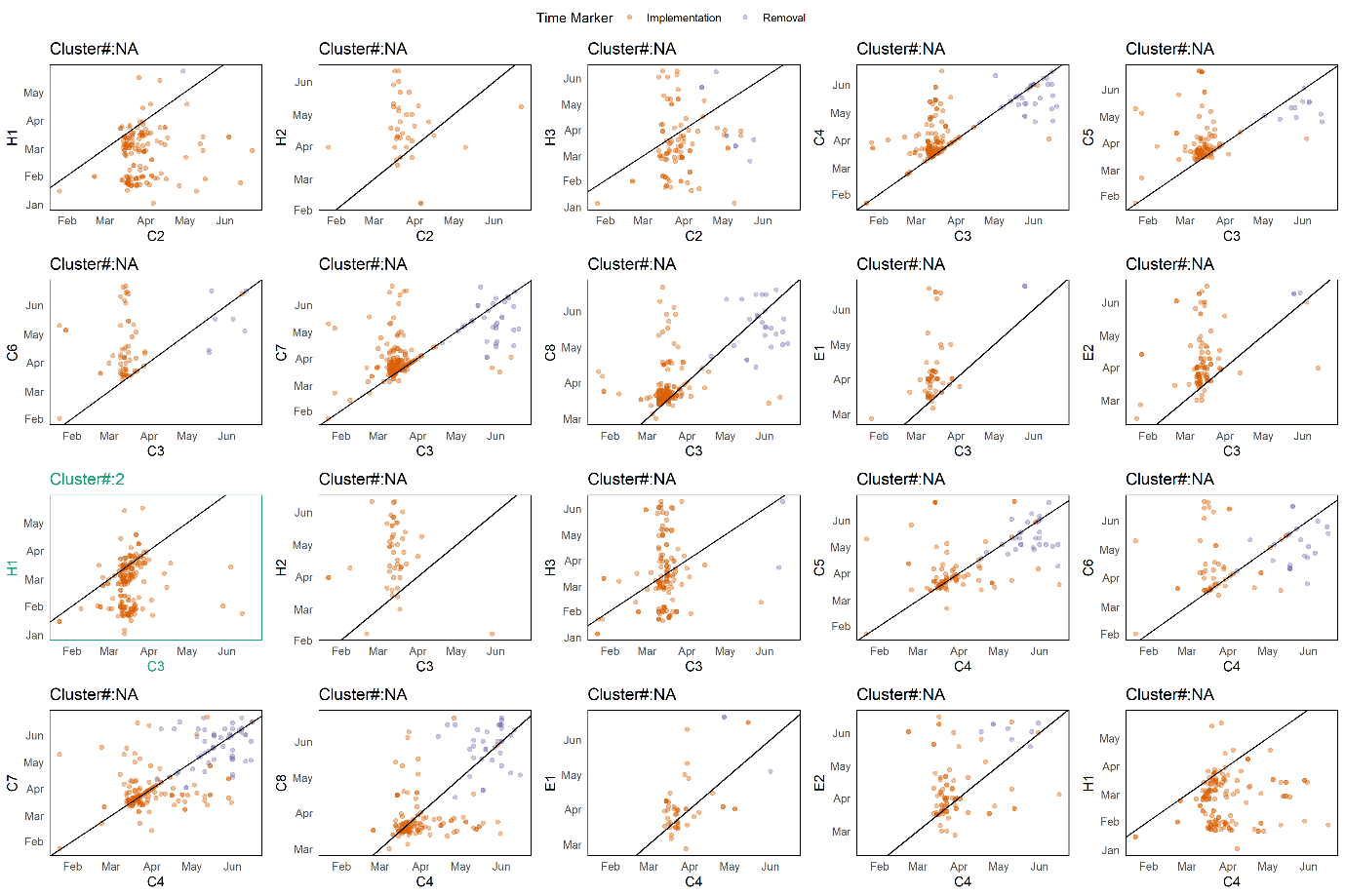
**

Figure S3 (continued).

***Maximum Effort Scenario***

**
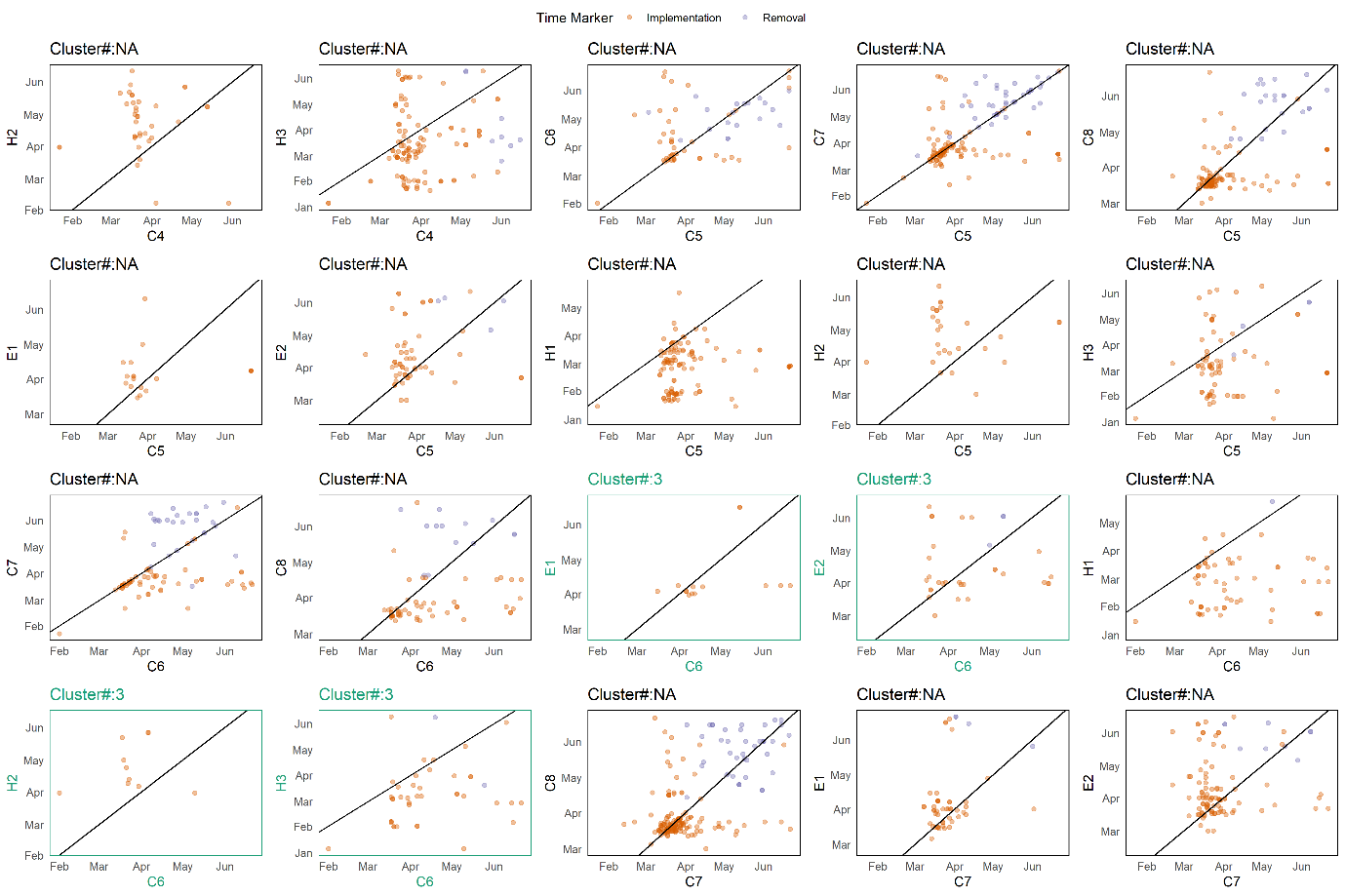
**

Figure S3 (continued).

***Maximum Effort Scenario***

**
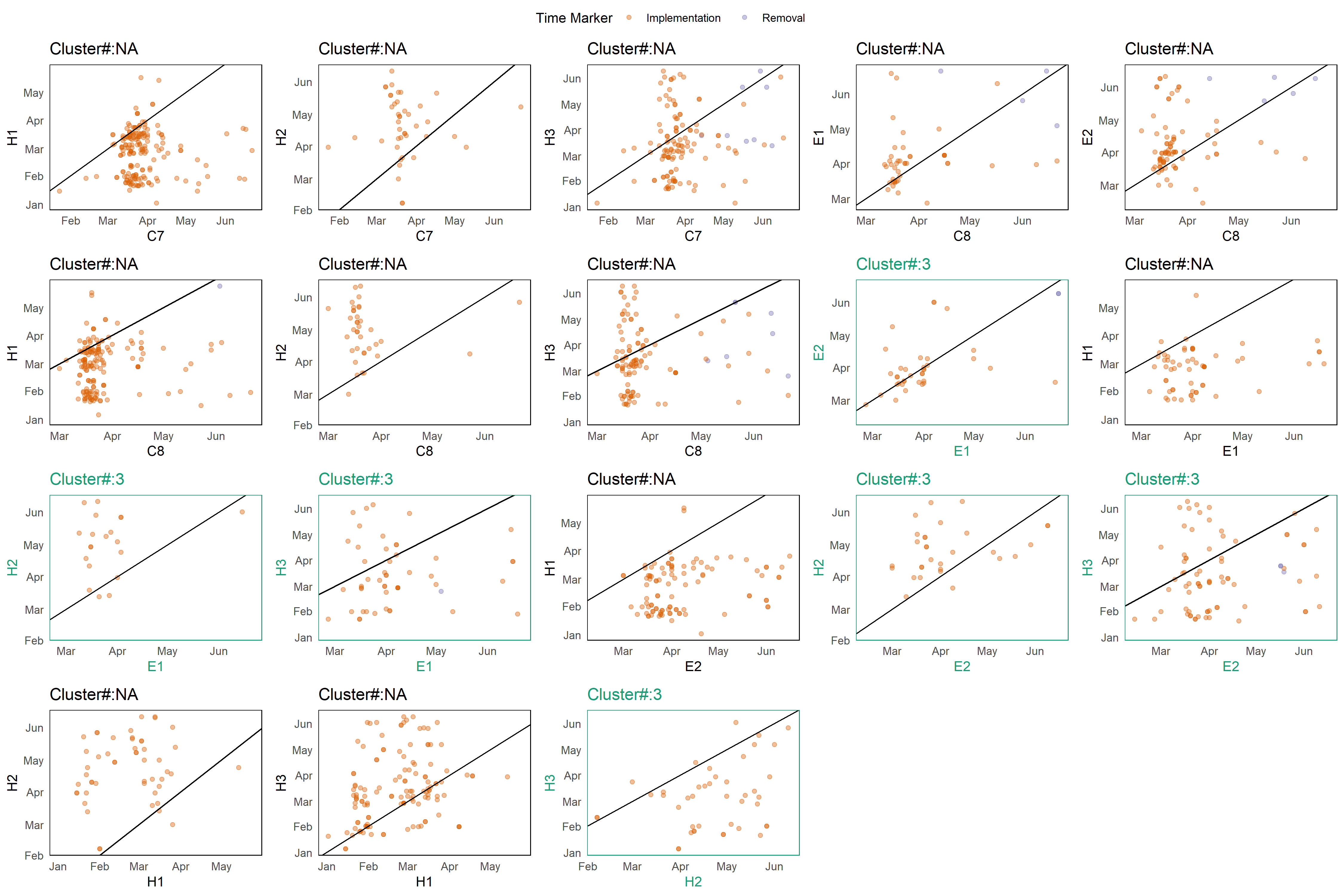
**

Figure S3 (continued).

# **A6. Temporal Lags**

As mentioned in Appendix 1, the original Oxford COVID-19 Government Response Tracker contains NPIs information in the format of ordinal categorical variables [3]. Take “Restrictions on Gatherings” for example, there are five levels:

0 - No restrictions;

1 - Restrictions on very large gatherings (the limit is above 1000 people)

2 - Restrictions on gatherings between 101-1000 people

3 - Restrictions on gatherings between 11-100 people

4 - Restrictions on gatherings of 10 people or less.

As discussed in the main text, we analysed two key scenarios:

- *Any effort scenario*: NPIs are binary variables, considered “present” as long as any (non-zero) effort is made;
- *Maximum effort scenario*: NPIs are binary variables, considered “present” only if the maximum effort is made.

For a hypothetical country’s, the NPI time-series regarding “Restrictions on Gathering” such as {0, 0, 0, 1, 1, 2, 3, 4, 4} is converted to:

- *Any effort scenario* as {0, 0, 0, 1, 1, 1, 1, 1, 1}
- *Maximum effort scenario* as {0, 0, 0, 0, 0, 0, 0, 1, 1}

An additional scenario - *multilevel effort scenario*, which preserves the original time series without conversion, was originally explored but had to be excluded due to severe temporal clustering and biased effect size estimates. However, here in the appendix we still showed some of these results, showcasing why they have not been included in the main discussion. For more details, please also see Appendix 5.


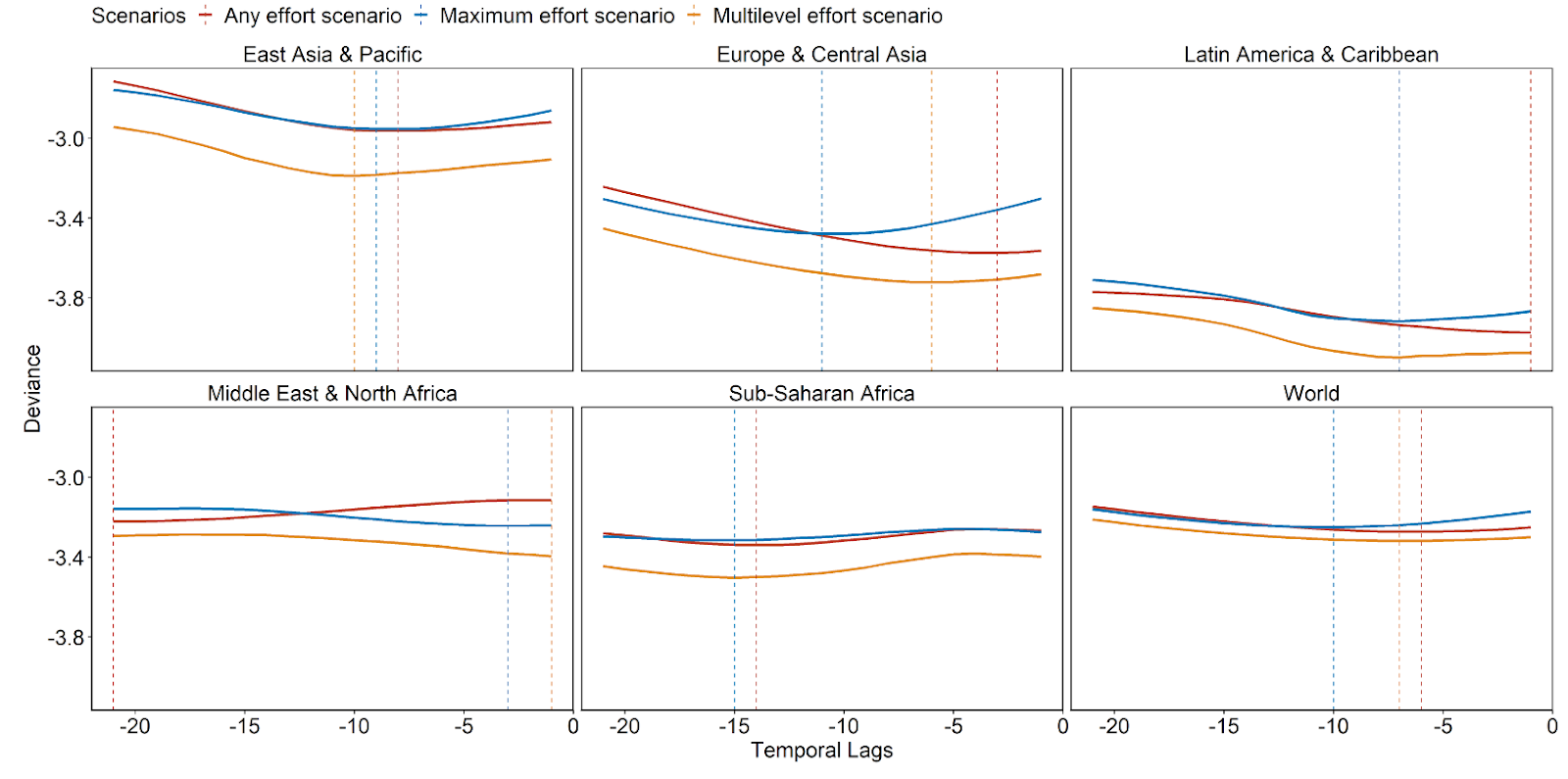


**Figure S4.** Deviance from panel analyses using different temporal lags between effective reproduction number and policy interventions. Models include all 13 interventions available. Deviance is defined as the logarithm of the sum of squared residuals divided by the number of data points. Full time-series from 1 Jan to 22 June 2020 were used. Dashed vertical lines indicate minimum deviances.

**
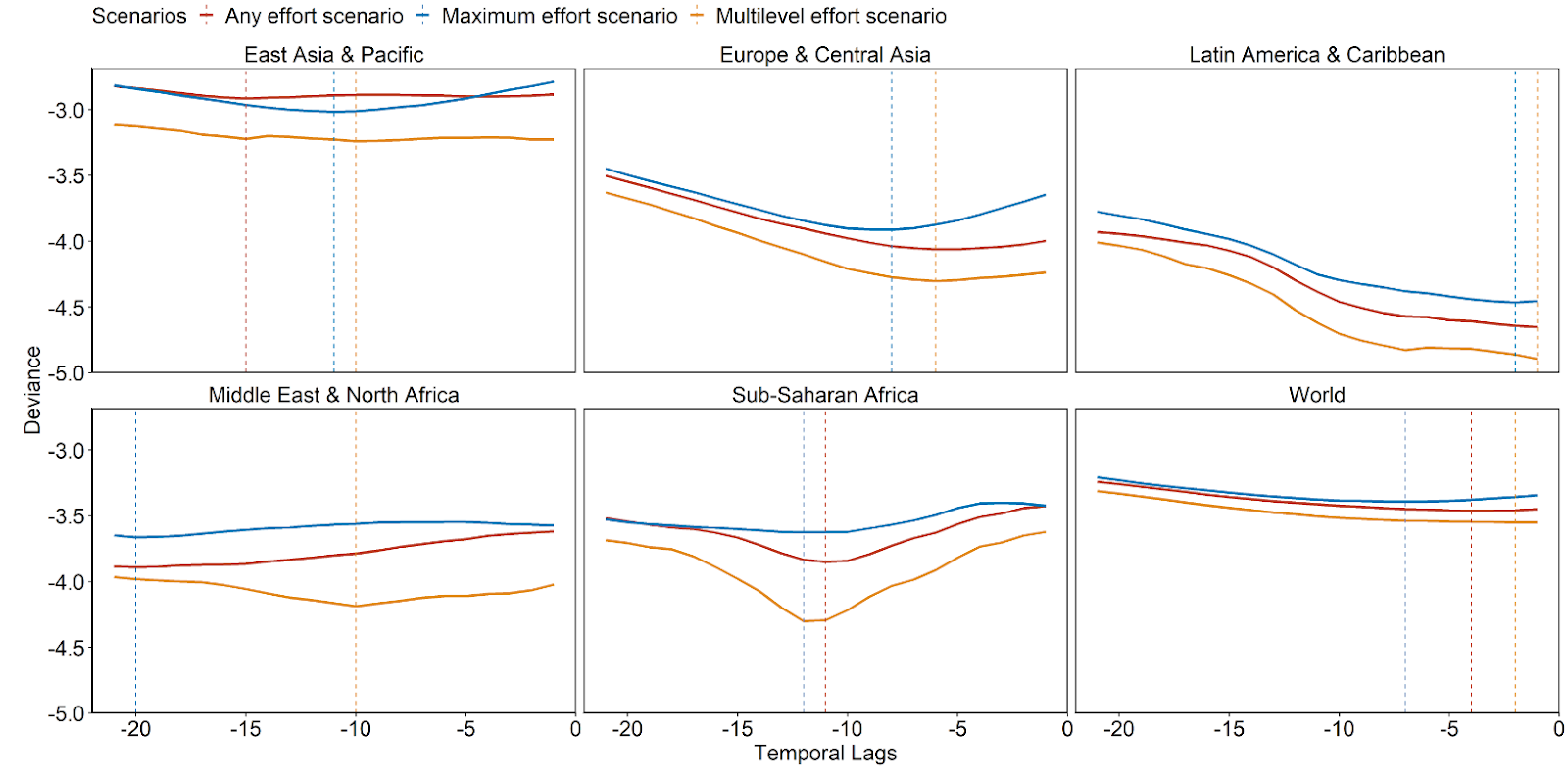
Figure S5.** Deviance from panel analyses using different temporal lags between effective reproduction number and policy interventions. Models include all 13 interventions available. Deviance is defined as the logarithm of the sum of squared residuals divided by the number of data points. Truncated time-series from 1 Jan to 13 April 2020 were used. The turning point on 13 April 2020 was identified using stringency indices. Dashed vertical lines indicate minimum deviances.

# **A7. Effect Validation Based on Univariable Panel Analyses**

# **
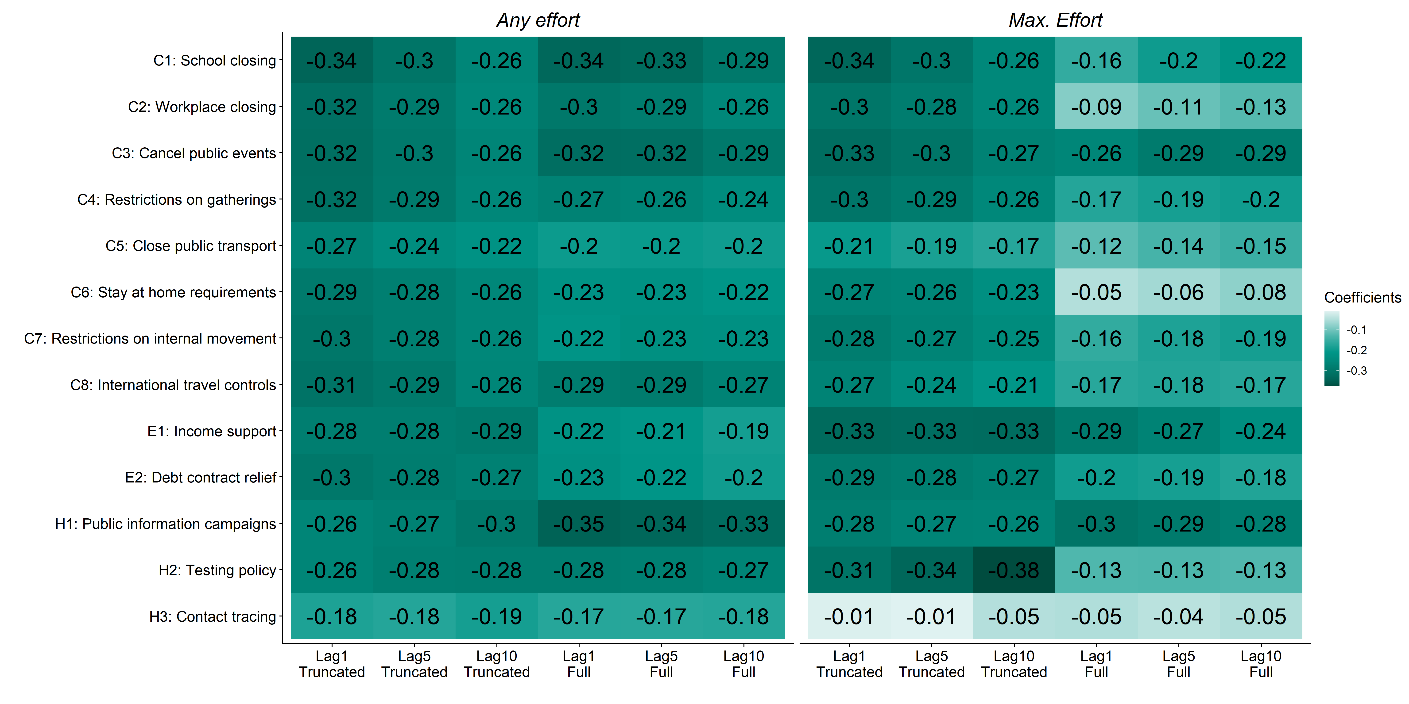
**

Figure S6. Univariable panel analyses – effect sizes.


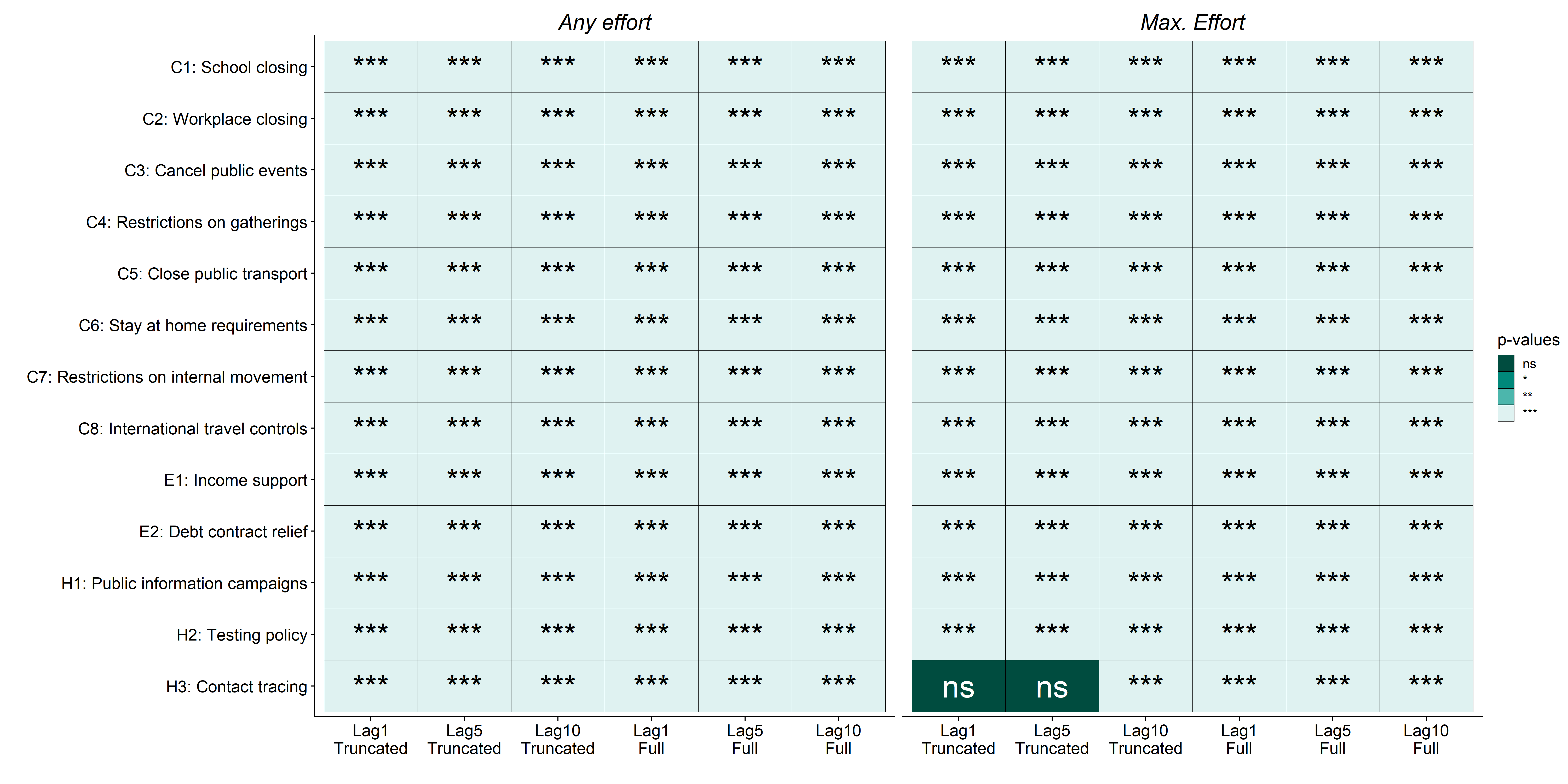


Figure S7. Univariable panel analyses – p-values.

# **A8. Model Fit by Country**

**Lowest Performance Country Fit**

|  | Country | Region | Average Rank | Average Mean Absolute Error |
| --- | --- | --- | --- | --- |
| 1 | Mongolia | East Asia & Pacific | 2.33 | 0.35 |
| 2 | Zambia | Sub-Saharan Africa | 3.21 | 0.35 |
| 3 | Zimbabwe | Sub-Saharan Africa | 6.83 | 0.29 |
| 4 | China | East Asia & Pacific | 7.67 | 0.28 |
| 5 | Chad | Sub-Saharan Africa | 7.75 | 0.29 |
| 6 | Uganda | Sub-Saharan Africa | 9.54 | 0.25 |
| 7 | Thailand | East Asia & Pacific | 9.58 | 0.27 |
| 8 | South Korea | East Asia & Pacific | 11.88 | 0.25 |
| 9 | Niger | Sub-Saharan Africa | 12.08 | 0.26 |
| 10 | Palestinian Territories | Middle East & North Africa | 13.13 | 0.23 |
| 11 | Djibouti | Middle East & North Africa | 15.33 | 0.27 |
| 12 | Sri Lanka | South Asia | 15.67 | 0.22 |
| 13 | Iceland | Europe & Central Asia | 16.75 | 0.24 |
| 14 | Qatar | Middle East & North Africa | 18.54 | 0.24 |
| 15 | South Sudan | Sub-Saharan Africa | 19.33 | 0.22 |
| 16 | Australia | East Asia & Pacific | 20.21 | 0.21 |
| 17 | Tanzania | Sub-Saharan Africa | 21.75 | 0.21 |
| 18 | Israel | Middle East & North Africa | 23.75 | 0.20 |
| 19 | Malawi | Sub-Saharan Africa | 24.67 | 0.20 |
| 20 | France | Europe & Central Asia | 25.96 | 0.18 |
| 21 | Venezuela | Latin America & Caribbean | 28.42 | 0.18 |
| 22 | Kuwait | Middle East & North Africa | 30.13 | 0.18 |
| 23 | Belarus | Europe & Central Asia | 31.38 | 0.17 |
| 24 | Peru | Latin America & Caribbean | 32.46 | 0.17 |
| 25 | Benin | Sub-Saharan Africa | 32.67 | 0.18 |
| 26 | Cameroon | Sub-Saharan Africa | 32.71 | 0.18 |
| 27 | New Zealand | East Asia & Pacific | 33.00 | 0.19 |
| 28 | Senegal | Sub-Saharan Africa | 33.50 | 0.17 |
| 29 | Bangladesh | South Asia | 34.79 | 0.16 |
| 30 | Italy | Europe & Central Asia | 35.79 | 0.17 |

**Table S5.** Lowest performance model fit by country.

**Highest Performance Country Fit**

|  | Country | Region | Average Rank | Average Mean Absolute Error |
| --- | --- | --- | --- | --- |
| 101 | Mali | Sub-Saharan Africa | 85.38 | 0.10 |
| 102 | Gabon | Sub-Saharan Africa | 85.50 | 0.09 |
| 103 | Kenya | Sub-Saharan Africa | 85.75 | 0.10 |
| 104 | Kyrgyzstan | Europe & Central Asia | 85.96 | 0.10 |
| 105 | Belgium | Europe & Central Asia | 87.92 | 0.09 |
| 106 | Oman | Middle East & North Africa | 89.54 | 0.09 |
| 107 | India | South Asia | 89.83 | 0.09 |
| 108 | Pakistan | South Asia | 90.17 | 0.09 |
| 109 | Cote d'Ivoire | Sub-Saharan Africa | 90.92 | 0.09 |
| 110 | Poland | Europe & Central Asia | 90.92 | 0.09 |
| 111 | Netherlands | Europe & Central Asia | 91.88 | 0.09 |
| 112 | Algeria | Middle East & North Africa | 92.67 | 0.09 |
| 113 | Panama | Latin America & Caribbean | 92.88 | 0.09 |
| 114 | Guinea | Sub-Saharan Africa | 93.33 | 0.08 |
| 115 | Cape Verde | Sub-Saharan Africa | 94.25 | 0.10 |
| 116 | Saudi Arabia | Middle East & North Africa | 95.88 | 0.09 |
| 117 | Cuba | Latin America & Caribbean | 98.29 | 0.08 |
| 118 | Puerto Rico | Latin America & Caribbean | 98.33 | 0.09 |
| 119 | Argentina | Latin America & Caribbean | 98.88 | 0.08 |
| 120 | Congo - Kinshasa | Sub-Saharan Africa | 99.50 | 0.08 |
| 121 | Suriname | Latin America & Caribbean | 99.75 | 0.09 |
| 122 | Indonesia | East Asia & Pacific | 99.88 | 0.08 |
| 123 | Brazil | Latin America & Caribbean | 101.79 | 0.08 |
| 124 | Dominican Republic | Latin America & Caribbean | 101.96 | 0.08 |
| 125 | Bolivia | Latin America & Caribbean | 102.46 | 0.08 |
| 126 | Mauritania | Sub-Saharan Africa | 110.50 | 0.09 |
| 127 | Romania | Europe & Central Asia | 111.96 | 0.06 |
| 128 | El Salvador | Latin America & Caribbean | 116.46 | 0.05 |
| 129 | Colombia | Latin America & Caribbean | 118.00 | 0.05 |
| 130 | Namibia | Sub-Saharan Africa | 129.92 | 0.04 |

**Table S6.** Highest performance model fit by country.

# **A9. Implementation Sequence of Non-Pharmaceutical Interventions**

A new ranking variable was created for each NPI in each scenario. For example, the NPI implemented first is ranked 1. The distributions of the sequential order for each NPI is shown below for both *any* and *maximum efforts* scenarios.

***Any Effort Scenario***


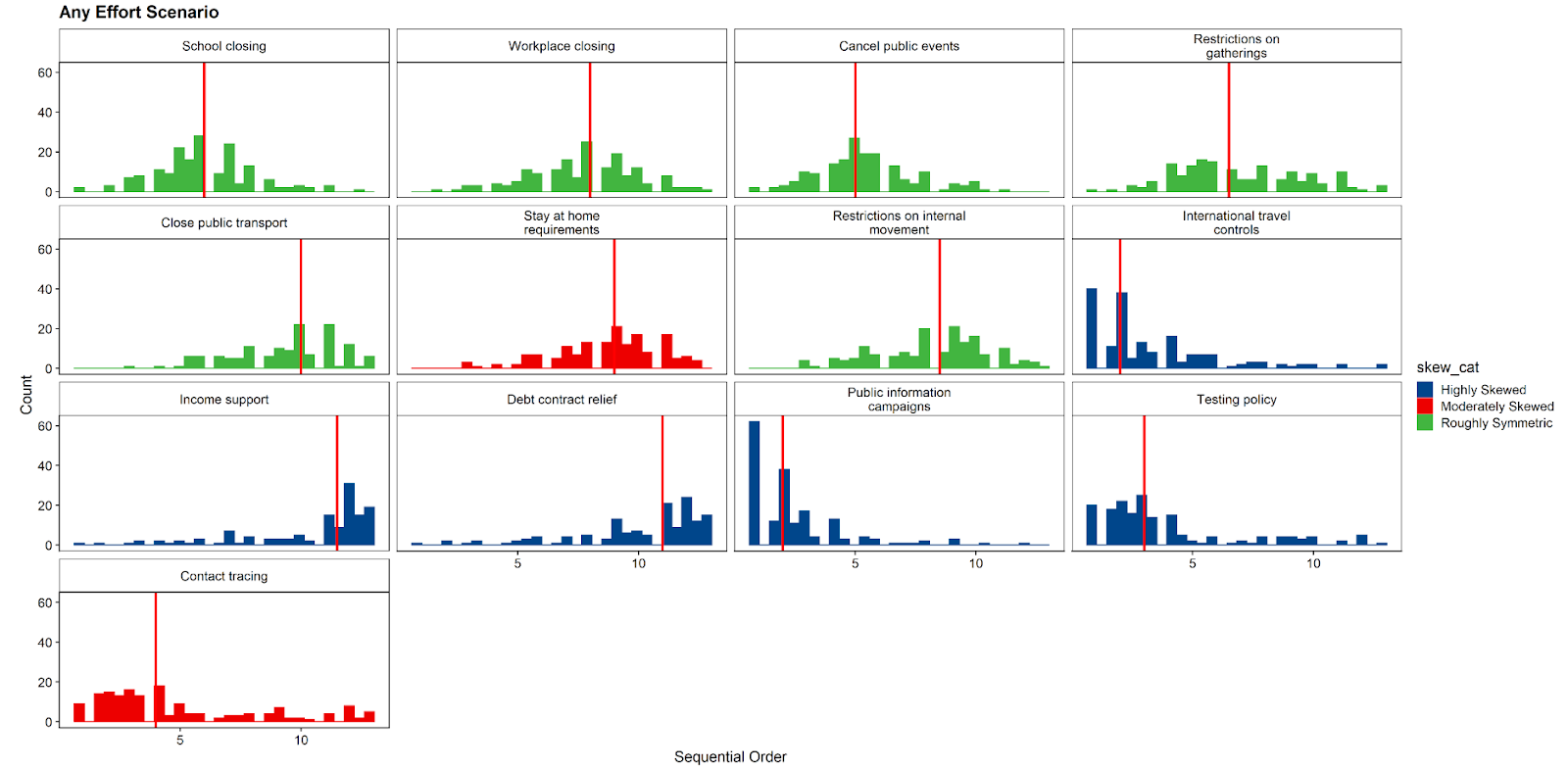


**Figure S8.** The sequential order of different NPIs under any effort scenario.

***Maximum Effort Scenario***


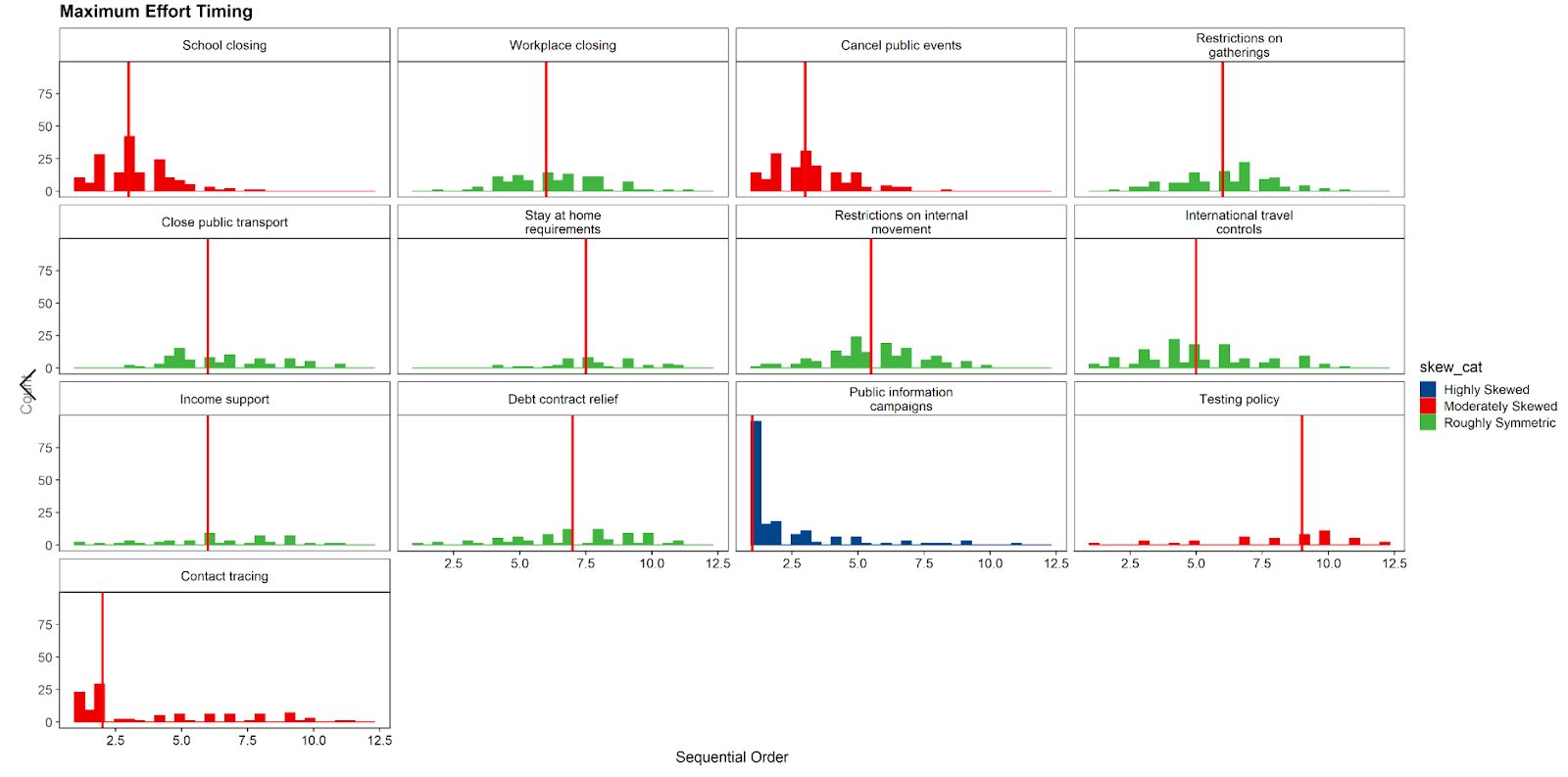


**Figure S9.** The sequential order of different NPIs under maximum effort scenario.

# **A10. Interpretation of Panel Analyses Results**

| NPI Code | Non-pharmaceutical Interventions | Any Efforts Scenario | Maximum Efforts Scenario |
| --- | --- | --- | --- |
| C1 | School closures | Strong | Strong |
| C2 | Workplace Closure | Strong | Weak |
| C3 | Public Events Cancellation | Weak | Strong |
| C4 | Restriction on gathering | Moderate | Strong |
| C5 | Public Transportation Closures | Weak | Weak |
| C6 | Stay-at-home requirements | Moderate | Weak |
| C7 | Internal Movement Restrictions | Strong | Strong |
| C8 | International Travel Controls | Weak | Weak |
| E1 | Income Support | Strong | Weak |
| E2 | Debt/ Contract Relief | Strong | Weak |
| H1 | Public Information Campaign | Weak | Moderate |
| H2 | Testing | Weak | Weak |
| H3 | Contact tracing | Weak | Weak |

**Table S7.** Statistical interpretation worksheets by YL and MJ detailing how these conclusions are reached can be downloaded from [<https://docs.google.com/spreadsheets/d/1lZUuxgXc6ZPCx4I_A56iYwokuDgU2if9nJySfDwqi50/edit?usp=sharing>].

# **A11. The *Multi-Level Scenario***

Besides the *any* and *maximum efforts scenarios* described in the main text, we also investigated if intermediate levels of NPIs led to any meaningful interpretation in terms of the impacts of NPIs. In this case, original data from the Oxford Government Response Tracker is preserved, with no additional conversion. We identify two temporal clusters that cover all 13 NPIs available for the analysis.
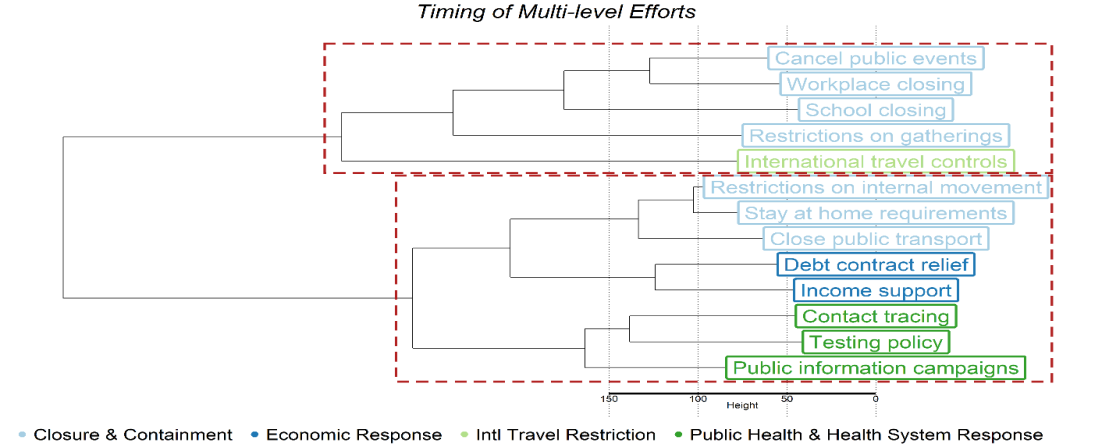


**Figure S10.** Hierarchical cluster analysis of NPIs time-series using the *multilevel scenario*. Blues and greens are used to describe the corresponding NPI groups; red boxes indicate statistically significant temporal clusters. Hierarchical clustering was carried out using Ward’s method; the statistical significance of temporal clusters identified was obtained via bootstrapping.


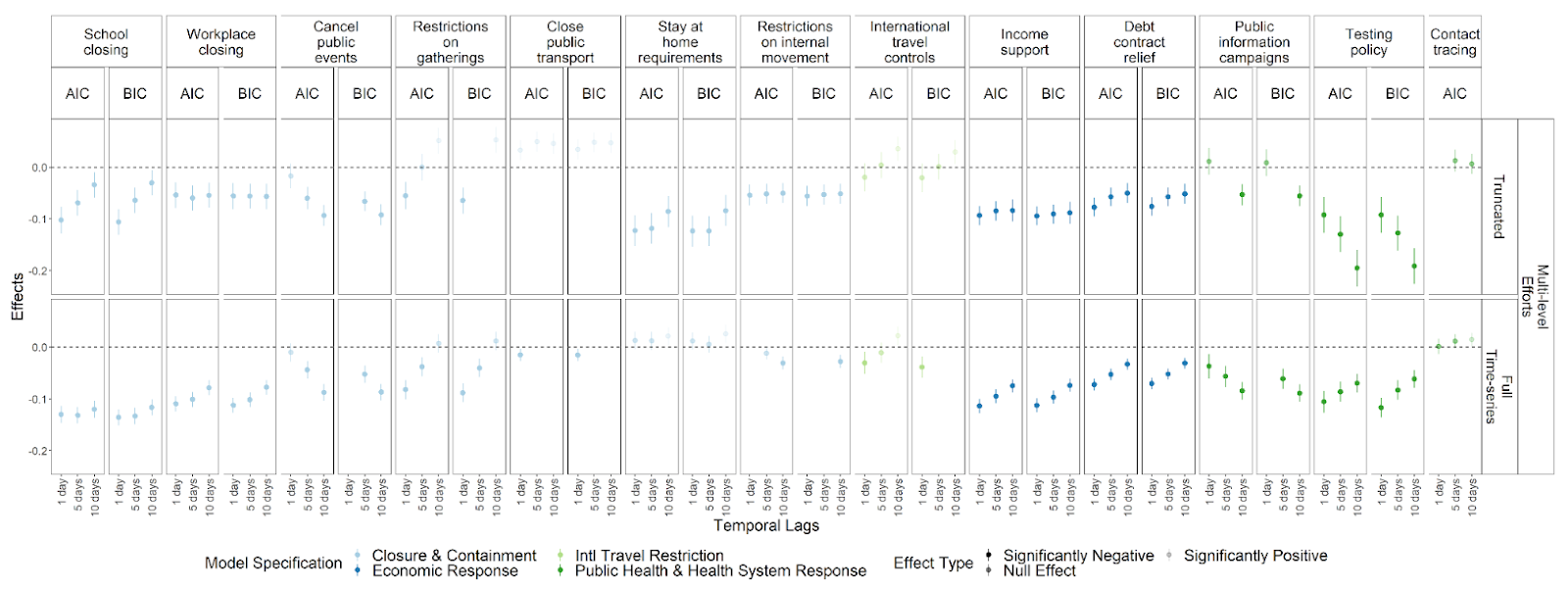


**Figure S11.** Effect sizes for each NPI from the selected models. Points and lines indicate mean and 95% confidence intervals. Upon examining the effect sizes, we identify at least two NPIs with positive effect estimates, which indicates estimates for all NPIs are susceptible to statistical bias, preventing us from drawing reasonable conclusions. Thus, we do not include this analysis in the discussion in the main text. Current data available does not seem to allow us to examine the impacts of NPIs to this level of details.

**A12. Related studies**

We searched PubMed and medRxiv on 25 July 2020 for articles including each of the following clusters of terms: (i) “covid” OR “coronavirus” OR “SARS-CoV-2”, (ii) “non-pharmaceutical” OR “distancing” OR “lockdown” OR “stay-at-home” OR “closure”, (iii) “transmission” OR “cases” OR “reproduction number”, and (iv) “model” or “regression”. We found 267 and 196 articles on PubMed and medRxiv respectively and supplemented them with an additional 4 articles from the reviewers’ knowledge and reference lists of articles we found. However, only 26 articles used real-world retrospective data from multiple countries to relate NPIs to SARS-CoV-2 transmission or a suitable proxy such as reproduction numbers, cases or deaths. Furthermore, 17 of these only examined the effect of a single intervention. Of the remaining nine, most of them did not adjust for temporal confounding (i.e. multiple NPIs occurring at similar times). A few took this into account by examining the number of NPIs that had already been implemented [11], excluding non-significant variables in scenario analysis [12] or grouping policies that were implemented in similar periods [13, 14].

| **First author** | **Source** | **Interventions** | **# of countries**  **/areas** | **Regions** | **Outcome** | **Type of model** | **Accounting for temporal confounding** |
| --- | --- | --- | --- | --- | --- | --- | --- |
| Alfano  [15] | PubMed | Lockdown | 202 | World | Reported cases | Interrupted time-series | No adjustment; single intervention only |
| Amer  [14] | PubMed | lockdown; loose lockdown; intermediate lockdown; strict lockdown; rapid testing increase, intermediate testing increase, slow testing increase | 6 | USA, Europe | Infected patient ratio | Bayesian hierarchical regression | No adjustment; interventions were divided into temporal stages |
| Banholzer [16] | Knowledge | 7 categories of NPIs | 20 | Europe, USA, Canada, Australia | Reported cases | Bayesian hierarchical model | No explicit adjustment; intervention effects fitted within the model framework |
| Bellali  [17] | medRxiv | Lockdown | 186 | World | Cases, deaths | Linear regression | No adjustment; single intervention only |
| Brauner [18] | Knowledge | 9 categories of NPIs | 41 | Mostly European | Rt inferred from cases and deaths | A Bayesian hierarchical model with global and country-specific effect parameters | No explicit adjustment; intervention effects fitted within the model framework |
| Chen  [12] | Knowledge | 6 categories of NPIs | 9 | World | Rt | Linear regression using output from the SIR model | Limited adjustment; exclusion of non-significant variables in scenario analysis |
| Chowell [19] | medRxiv | Intervention | 5 | USA, Europe | Cases | Logistic growth | No adjustment; single intervention only |
| Rey  [20] | medRxiv | Testing strategy | 8 | SAARC countries | Cases, deaths | SIR model | No adjustment; qualitative prediction only |
| Flaxman [21] | PubMed | Lockdown, public events cancellation, school closure, self-isolation, social distancing | 11 | Europe | Rt inferred from deaths with time lags | Bayesian hierarchical model | No explicit adjustment; intervention effects fitted within the model framework |
| Ghosal  [22] | PubMed | Lockdown | 12 | Europe, Asia, New Zealand | Weekly change in reported cases and deaths | Fixed effect panel analysis model | No adjustment; single intervention only |
| Haug  [11] | medRxiv | 3850 categories of NPIs | 76 | World | Rt | Machine learning | Examine the number of NPIs that countries have already implemented |
| Hsiang  [13] | Knowledge | More than 1000 NPIs | 6 | China, South Korea, Italy, Iran, France, USA | Active cases | Panel regression using output from the SIR model | Adjustment by policy grouping |
| Jüni  [23] | PubMed | Restrictions of mass gathering; social distancing; school closures | 144 | World | The ratio of rate ratio between follow-up period and exposure period | Wallinga-Lipsitch model | No adjustment |
| Karnakov [24] | medRxiv | "Intervention" (social distancing) | 51 | Europe | R0 | SIR model | No adjustment; single intervention only |
| Linka  [25] | PubMed | Travel restrictions | 27 | Europe | Cases | SEIR model | No adjustment; single intervention only |
| Liu  [26] | medRxiv | Lockdown | 2 | Wuhan, New York | Cases | Individual-based SIR model | No adjustment; single intervention only |
| Lonergan [27] | PubMed | Lockdown | 73 | World | R calculated from Reported cases; Deaths | Random effect regression | No adjustment; single intervention only |
| López  [28] | PubMed | Lockdown | 4 | USA, Indonesia, Argentina, Japan | Epidemic characteristics (e.g., peak timing and peak size) | There's no formal analysis, they just calculated the IPR for different countries before and after, and voila! | No adjustment; single intervention only |
| McGrail [29] | medRxiv | "Social distancing" | 26 | World | Cases | Linear regression | No adjustment; single intervention only |
| Mishra  [30] | medRxiv | Non-specific; compared growth rates between countries | 23 | World | Cases | Exponential equation | No adjustment |
| Osherovich [31] | medRxiv | "Social distancing" | 2 | Italy, Spain | Cases, deaths | Double power law equation | No adjustment; single intervention only |
| Petr  [32] | PubMed | Lockdown | 51 | Europe | Rt | Hierarchical cluster linear regression | No adjustment; single intervention only |
| Sinha  [33] | medRxiv | Testing intensity | 98 | World | Cases, deaths | Linear regression | No adjustment; single intervention only |
| Tobías  [34] | PubMed | Lockdown | 2 | Europe | Reported cases; Deaths; ICU admissions | Stochastic SIR model | No adjustment; single intervention only |
| Wang  [35] | medRxiv | Non-specific; compared growth rates at different times | 4 | China, South Korea, Italy, USA | Cases | Survival-convolution model | No adjustment |
| Yang  [36] | medRxiv | Surveillance, suppression, isolation and hybrid | 2 | Wuhan, London | Cases | SEIR model | No adjustment; based on differences between cities and phases |
| Li  [37] | PubMed | School closure, workplace closure, public events ban, ban of gathering, public transportation closure, stay-at-home requirement, internal movement limits, international travel limits | 131 | World | Rt | Log-linear regression model | No adjustment |

**Table S8.** Review of existing literature.

**Reference**

Index based on the main text.

3. Hale T, Angrist N, Kira B, Petherick A, Phillips T, Webster S. Variation in government responses to COVID-19. BSG-WP-2020/032. Version 5.0. [Internet]. 2020 [cited 2020 May 11]. Available from: https://www.bsg.ox.ac.uk/sites/default/files/2020-05/BSG-WP-2020-032-v5.0_0.pdf

11. Haug N, Geyrhofer L, Londei A, Dervic E, Desvars-Larrive A, Loreto V, et al. Ranking the effectiveness of worldwide COVID-19 government interventions. medRxiv. 2020 Jul 8;2020.07.06.20147199.

12. Chen X, Qiu Z. Scenario analysis of non-pharmaceutical interventions on global COVID-19 transmissions. arXiv:200404529 [physics, q-bio, stat] [Internet]. 2020 Apr 15 [cited 2020 Aug 10]; Available from: http://arxiv.org/abs/2004.04529

13. Hsiang S, Allen D, Annan-Phan S, Bell K, Bolliger I, Chong T, et al. The effect of large-scale anti-contagion policies on the COVID-19 pandemic. Nature. 2020 Jun 8;1–9.

14. Amer F, Hammoud S, Farran B, Boncz I, Endrei D. Assessment of Countries’ Preparedness and Lockdown Effectiveness in Fighting COVID-19. Disaster Medicine and Public Health Preparedness. undefined/ed;1–8.

15. Alfano V, Ercolano S. The Efficacy of Lockdown Against COVID-19: A Cross-Country Panel Analysis. Appl Health Econ Health Policy. 2020 Aug 1;18(4):509–17.

16. Banholzer N, Weenen E van, Kratzwald B, Seeliger A, Tschernutter D, Bottrighi P, et al. Impact of non-pharmaceutical interventions on documented cases of COVID-19. medRxiv. 2020 Apr 28;2020.04.16.20062141.

17. Bellali H, Chtioui N, Chahed M. Factors associated with country-variation in COVID-19 morbidity and mortality worldwide: an observational geographic study | medRxiv. medRxiv [Internet]. [cited 2020 Aug 13]; Available from: https://www.medrxiv.org/content/10.1101/2020.05.27.20114280v1

18. Brauner JM, Mindermann S, Sharma M, Stephenson AB, Gavenčiak T, Johnston D, et al. The effectiveness and perceived burden of nonpharmaceutical interventions against COVID-19 transmission: a modelling study with 41 countries. medRxiv. 2020 Jun 2;2020.05.28.20116129.

19. Chowell G, Rothenberg R, Roosa K, Tariq A, Hyman JM, Luo R. Sub-epidemic model forecasts for COVID-19 pandemic spread in the USA and European hotspots, February-May 2020. medRxiv. 2020 Jul 4;2020.07.03.20146159.

20. Rey SK, Rahman MdM, Shibly KH, Siddiqi UR, Howlader A. Epidemic Trend Analysis of SARS-CoV-2 in SAARC Countries Using Modified SIR (M-SIR) Predictive Model | medRxiv. medRxiv [Internet]. [cited 2020 Aug 13]; Available from: https://www.medrxiv.org/content/10.1101/2020.06.29.20142513v1

21. Flaxman S, Mishra S, Gandy A, Unwin HJT, Mellan TA, Coupland H, et al. Estimating the effects of non-pharmaceutical interventions on COVID-19 in Europe. Nature. 2020 Jun 8;1–8.

22. Ghosal S, Bhattacharyya R, Majumder M. Impact of complete lockdown on total infection and death rates: A hierarchical cluster analysis. Diabetes Metab Syndr. 2020;14(4):707–11.

23. Jüni P, Rothenbühler M, Bobos P, Thorpe KE, Costa BR da, Fisman DN, et al. Impact of climate and public health interventions on the COVID-19 pandemic: a prospective cohort study. CMAJ. 2020 May 25;192(21):E566–73.

24. Karnakov P, Arampatzis G, Kičić I, Wermelinger F, Wälchli D, Papadimitriou C, et al. Data driven inference of the reproduction number (R0) for COVID-19 before and after interventions for 51 European countries. medRxiv. 2020 May 23;2020.05.21.20109314.

25. Linka K, Peirlinck M, Costabal FS, Kuhl E. Outbreak dynamics of COVID-19 in Europe and the effect of travel restrictions. Computer Methods in Biomechanics and Biomedical Engineering. 2020 May 5;0(0):1–8.

26. Liu X. A Simple, SIR-like but Individual-Based l-i AIR Model: Application in Comparison of COVID-19 in New York City and Wuhan. medRxiv. 2020 Jun 2;2020.05.28.20115121.

27. Lonergan M, Chalmers JD. Estimates of the ongoing need for social distancing and control measures post-“lockdown” from trajectories of COVID-19 cases and mortality. European Respiratory Journal [Internet]. 2020 Jul 1 [cited 2020 Aug 10];56(1). Available from: https://erj.ersjournals.com/content/56/1/2001483

28. López L, Rodó X. The end of social confinement and COVID-19 re-emergence risk. Nature Human Behaviour. 2020 Jul;4(7):746–55.

29. McGrail DJ, Dai J, McAndrews KM, Kalluri R. Enacting national social distancing policies corresponds with dramatic reduction in COVID19 infection rates. medRxiv. 2020 Apr 29;2020.04.23.20077271.

30. Mishra PK, Mishra S. A deductive approach to modeling the spread of COVID-19. medRxiv. 2020 Mar 30;2020.03.26.20044651.

31. Osherovich VA, Fainberg J, Osherovich LZ. DOUBLE POWER LAW FOR COVID-19: PREDICTION OF NEW CASES AND DEATH RATES IN ITALY AND SPAIN. medRxiv. 2020 May 11;2020.05.07.20094714.

32. Petr K, Georgios A, Fabian W, Daniel W, Costas P, Petros K. Data-driven inference of the reproduction number for COVID-19 before and after interventions for 51 European countries. Swiss Medical Weekly [Internet]. [cited 2020 Aug 13]; Available from: https://smw.ch/article/doi/smw.2020.20313

33. Ghosal S, Sinha B, Sengupta S, Majumder M. Frequency of testing for COVID 19 infection and the presence of higher number of available beds per country predict outcomes with the infection, not the GDP of the country - A descriptive statistical analysis. medRxiv. 2020 Apr 6;2020.04.01.20047373.

34. Tobías A. Evaluation of the lockdowns for the SARS-CoV-2 epidemic in Italy and Spain after one month follow up. Science of The Total Environment. 2020 Jul 10;725:138539.

35. Wang Q, Xie S, Wang Y, Zeng D. Survival-Convolution Models for Predicting COVID-19 Cases and Assessing Effects of Mitigation Strategies. medRxiv [Internet]. [cited 2020 Aug 13]; Available from: https://www.medrxiv.org/content/10.1101/2020.04.16.20067306v2

36. Yang P, Qi J, Zhang S, Wang X, Bi G, Yang Y, et al. Feasibility Study of Mitigation and Suppression Intervention Strategies for Controlling COVID-19 Outbreaks in London and Wuhan. medRxiv. 2020 Apr 15;2020.04.01.20043794.

37. Li Y, Campbell H, Kulkarni D, Harpur A, Nundy M, Wang X, et al. The temporal association of introducing and lifting non-pharmaceutical interventions with the time-varying reproduction number (R) of SARS-CoV-2: a modelling study across 131 countries. The Lancet Infectious Diseases [Internet]. 2020 Oct 22 [cited 2020 Nov 4];0(0). Available from: https://www.thelancet.com/journals/laninf/article/PIIS1473-3099(20)30785-4/abstract
